# Age-dependent changes in mean and variance of gene expression across tissues in a twin cohort

**DOI:** 10.1093/hmg/ddx424

**Published:** 2017-12-08

**Authors:** Ana Viñuela, Andrew A Brown, Alfonso Buil, Pei-Chien Tsai, Matthew N Davies, Jordana T Bell, Emmanouil T Dermitzakis, Timothy D Spector, Kerrin S Small

**Affiliations:** 1 Department of Twin Research and Genetic Epidemiology, King's College London, St Thomas' Campus, SE1 7EH London, UK; 2 Department of Genetic Medicine and Development, University of Geneva Medical School, 1211 Geneva, Switzerland; 3 Institute for Genetics and Genomics in Geneva (iGE3), University of Geneva, 1211 Geneva, Switzerland; 4 Swiss Institute of Bioinformatics, 1211 Geneva, Switzerland; 5 Wellcome Trust Sanger Institute, Hinxton CB10 1SA, Cambridge, UK; 6 Division of Mental Health and Addiction, NORMENT, KG Jebsen Centre for Psychosis Research, Oslo University Hospital, Oslo 0450, Norway

## Abstract

Changes in the mean and variance of gene expression with age have consequences for healthy aging and disease development. Age-dependent changes in phenotypic variance have been associated with a decline in regulatory functions leading to increase in disease risk. Here, we investigate age-related mean and variance changes in gene expression measured by RNA-seq of fat, skin, whole blood and derived lymphoblastoid cell lines (LCLs) expression from 855 adult female twins. We see evidence of up to 60% of age effects on transcription levels shared across tissues, and 47% of those on splicing. Using gene expression variance and discordance between genetically identical MZ twin pairs, we identify 137 genes with age-related changes in variance and 42 genes with age-related discordance between co-twins; implying the latter are driven by environmental effects. We identify four eQTLs whose effect on expression is age-dependent (FDR 5%). Combined, these results show a complicated mix of environmental and genetically driven changes in expression with age. Using the twin structure in our data, we show that additive genetic effects explain considerably more of the variance in gene expression than aging, but less that other environmental factors, potentially explaining why reliable expression-derived biomarkers for healthy-aging have proved elusive compared with those derived from methylation.

## Introduction

Aging is a complex process, characterized by a progressive decline in an organism's biological function and change in phenotypic characteristics, which leads to an increased chance of developing disease and ultimately the death of the organism (1). Others have attempted to understand the aging process by identifying common denominators of aging in different organisms (2). Many of these hallmarks, such as genome instability, epigenetic alterations, loss of proteostasis and telomere attrition, are accompanied by changes in gene expression. Identification of genes that are differentially expressed with age has proved useful in identifying pathways whose behavior is modified by age, as well as identifying biomarkers of aging and therapeutic targets (3–5). Expression studies into aging using animal models have discovered that the expression of up to 75% of genes can be associated with aging (6). These modifications can occur by acting on the level of expression of genes, on the splicing of the mRNA produced or on the genetic regulation of gene expression (6,7). Human studies recently managed to identify thousands of genes associated with age in multiple tissues (3,8,9), but have not observed age effects with the same scale and diversity as those seen in model organisms. Reasons for this include a reduced power to see interactions due to the uncontrolled human environment and inbred nature of model organisms.

In this study, we investigate changes in gene expression with age using RNA-seq measurements of fat, skin, whole blood and derived lymphoblastoid cell lines (LCLs) expression from 855 monozygous (MZ) and dizygous (DZ) adult female twins (Supplementary Material, Table S1). We take a comprehensive approach that includes not only an analysis of the effect of age on the mean of gene expression and alternative splicing, but also analyses using gene expression variance and discordance between genetically identical MZ twin pairs. We aim with this to better understand the relationship between age-dependent changes in phenotypic variance, a decline in regulatory functions with age and the subsequent increase in disease risk observed in epidemiological studies. The age-related changes in variance of gene expression have been identified in animal models, but identification of specific genes changing variance with age in humans has not been well studied (10–13). We show also how environmental exposures on MZ siblings change expression over time and how the aging process is a complicated interplay between genetic variance and environmental factors. To explore one possible environmental factor involved, we also studied methylation changes with age in the same samples from fat tissue and genotype-by-age interaction on gene expression. Finally, in comparison with previous studies, we observe a greater degree of sharing of age expression effects across tissues, reflecting the large sample, benefits of the twin design and the more accurate quantification provided by RNA-seq.

## Results

### Effects of aging on levels of gene expression

**Figure 1.**
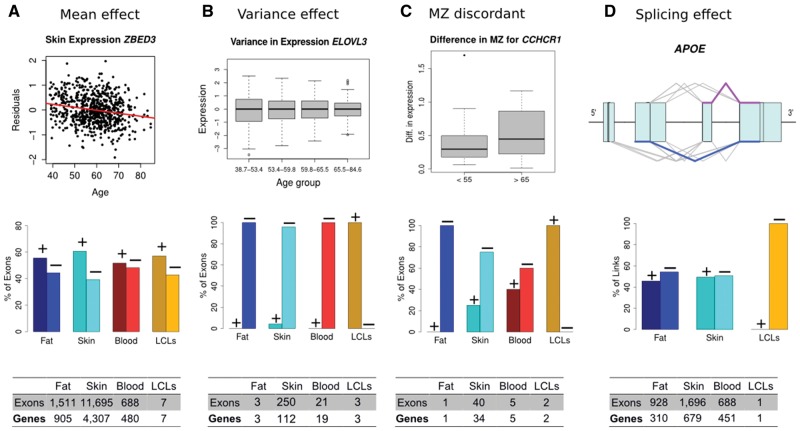
Effects of aging in gene expression: The effect of aging in gene expression is not limited to changes in mean expression values with age (**A**), but includes also changes in levels of phenotypic variance (**B**, **C**), and splicing (**D**). The top row graphs show real data examples for the effects of aging in expression investigated. The middle graphs show bar plots with the percentage of exons with positive (+) or negative (−) age effects in each analysis. And finally, the bottom tables provide the number of exons and genes with significant association for each of the effects presented. All the real examples are from skin, the tissue with larger age effect in expression overall analyses. (A) Effect of aging in mean gene expression, usually referred as differentially expression with age in exons. The example shows the residuals (after removing technical covariates) of the expression of the *ZBED3* gene decreasing with age. Skin is the tissues with a larger effect of age in expression and LCLs the smaller. (B) The effect of aging in variance of gene expression is shown with the *ELOVL3* gene and a significant decrease of variance in expression with age. From the bar plot it is possible to appreciate that the majority of the significant exons had a decrease in variance with age. (C) Differences in expression between monozygous (MZ) twins point out to environmental factors different among the siblings affecting gene expression, since MZ twins are genetically identical individuals with the same age. The example shows the difference in expression between MZ twins in the gene *CCHR1*. (D) For the splicing analysis, only links (reads between two exons) were considered. The example shows the structure of the gene *APOE* with its exons (boxes) and lines connecting the exons representing reads spanning between two exons. The number of reads linking exons 3 and 4 (in purple) decreased in number with age, while reads linking exons 2 and 4 (blue) increased with age (Fig. 5 for details). The model suggested that an isoform skipping the third exon (from the 5') may be more abundant in older individuals compare to an isoform that includes the third exon linked to the last exon.

The analysis of changes in gene expression with age used RNA-seq data from 855 healthy individuals drawn from the TwinsUK cohort (Supplementary Material, Table S1) in four tissues: i) photo protected skin, ii) subcutaneous fat, iii) whole blood and iv) lymphoblastoid cell lines (LCLs). We consider a gene associated with age if at least one exon was associated with chronological age. We discovered that 36.6% of tested genes (5631 of 15 353) had at least one exon where expression was significantly associated with age in at least one tissue (adjusted *P* value < 0.05, the multiple testing procedure used controls for both the number of genes and the differing number of exons per gene); (Fig. 1A, Supplementary Material, File S1, Supplementary Material, Table S2). This number is roughly double that we previously reported (18.3%, 3019 genes) using exactly the same skin, fat and LCLs samples but measuring expression using microarrays (3) (Fig. 1A, Supplementary Material, Fig. S1). Our results increase the current catalogue of genes which expression changes with age and the list of potential biomarkers of aging.

### Effects of aging on splicing

As well as changes in the average level of expression, age is also known to cause changes in the mRNA splicing process. To identify changes in splicing with age, we produced quantifications of this process using Altrans (14), which considers links between reads. We found a total of 904 genes (6.3% of the 14, 261 genes with more than one exon expressed) with at least one of their links spanning from two exons differentially expressed with age (adjusted *P* values < 0.05, Supplementary Material, Files S2 and S3). Differential splicing was only observed in fat and skin; possibly because of the low power due to sample size in blood and the LCL transformation process removing environmental effects. We see fewer genes with differential splicing compared with differential expression, but this could be because of the increased difficulty in producing a reliable quantification of the splicing process using current technologies. In the future, quantifications based on long read sequencing will allow accurate quantification of isoforms; revisiting splicing in this context may dramatically alter our perspective.

### Additive genetic effects explain a substantially larger amount of variance in gene expression than age in all tissues

**Figure 2.**
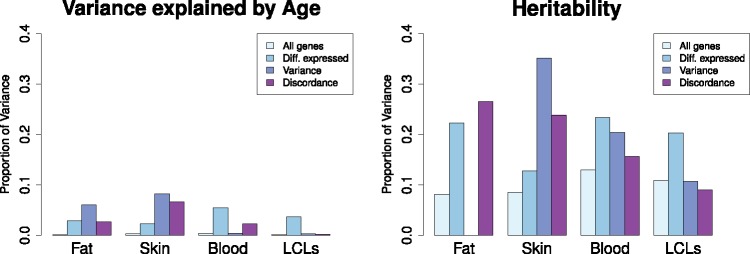
Median proportion of variance explained by age (left) and genetics (right) in all tested genes (All genes), differentially expressed genes with age (diff. expressed), genes changing variance with age (Variance) and genes discordant in MZ twins with age (Discordant). In general, the amount of variance explained by age and heritability in genes significantly affected by age in different ways is larger than in the median of the whole genome. The exception applies to those groups of genes with very little number of genes, like discordance genes in fat with 1 gene. The complete variance decomposition analysis is shown in Supplementary Material, Table S4.

To quantify the relative effect of age and additive genetic effects on gene expression, we estimated the proportion of variance of exon expression levels (removing technical confounders) explained by age and additive genetic effects (heritability). In exons associated with age, age explained only a small proportion of the variation in gene expression, with median values between 2.2% and 5.7% depending on tissue and with maximum values ranging from 12% to 27% (Supplementary Material, File S4). Globally, the effect of age on expression was greatest in blood, then skin, fat, and finally LCLs had the least. In comparison, the proportion of variance explained by additive genetic effects on the same set of age-affected exons was greater than that explained by age in all tissues (median *h*^2^_skin_ = 0.12, *h*^2^_fat_ = 0.22, *h*^2^_LCLs_ = 0.20, *h*^2^_blood_ = 0.23, Fig. 2, Supplementary Material, Table S3 and Figs S2–S5). We also investigated heritability separately in older and younger individuals but observed no clear pattern (Supplementary Material, Table S4). These results combined demonstrate that though the ageing process impacts the expression of thousands of genes, in most cases this impact is relatively small compared with the impact of genetic variation on expression. This shows that while the consequences of ageing are global and widespread, it has low power for predicting expression. This suggests that a model of biological ageing based on expression, as have been proposed using methylation, may be difficult to produce.

### Variance and differences in gene expression between MZ twins are dependent on age

Age-dependent changes in the variance of gene expression (rather than mean expression levels) has been reported in different model organisms (10–12), but previous studies in humans have not been conclusive (13). Here, we looked for changes in variance with age and identified 137 genes where expression showed age-dependent variance in at least one tissue (adjusted *P* value < 0.05, Fig. 1B, Supplementary Material, File S5). Since changes in phenotypic variance have mainly been reported to increase with age, we were surprised to observe that for the majority of these genes we report a decrease in variance of expression with increasing age. The biological functions associated with the genes with age-associated differential variance in skin included oxidation reduction, with affected genes such as *SOD2* (Fig. 1B), fatty acid metabolism with genes including *CPT1B*, *ELOVL3* and *ELOVL5* or cell cycle control like *CDKN1A (p21)* (Supplementary Material, Fig. S6). Our analysis shows concrete examples of age-related changes in phenotypic variance affecting expression in humans and identified changes in variance with age as another process by which aging may be linked to disease.

Changes in the phenotypic variance with age can be due to different responses to environment, age-related damage accumulation leading to stochastic deregulation of gene expression or gene-age interactions where changes in relative genetic effects can increase heterogeneity across the population at a particular age (15). Since MZ twins are genetically identical (and the same age), differences in expression levels within twin-pairs must have an environmental cause, allowing us to learn whether changes in variance with age were indeed influenced by the environment experienced by the twins. Therefore, and exploiting the twin design, we calculated the difference in expression between MZ co-twins (Supplementary Material, Table S1). Here, we identified 42 genes where difference (discordance) in expression between MZ co-twins changed with age in at least one tissue (Fig. 1C, Supplementary Material, Fig. S7 and File S6). Of the 34 genes identified in skin, 14 also showed a change in variance with age. This indicates that the observed change for those genes was environmentally and not genetically driven (Supplementary Material, Fig. S8). However, for the 81 remaining genes, significant change in variance with age did not produce age-related discordance, either because the changing environments were shared by MZ twins, or the variance effect was due to GxE interactions. We conclude that changes in phenotypic variation with age can be attributed to different environmental exposures among the individuals and not only to a general decline in regulatory functions and increase in genome damage with age, as others have suggested (12).

### Genetic and environmental changes affecting variance in gene expression with age

**Figure 3.**
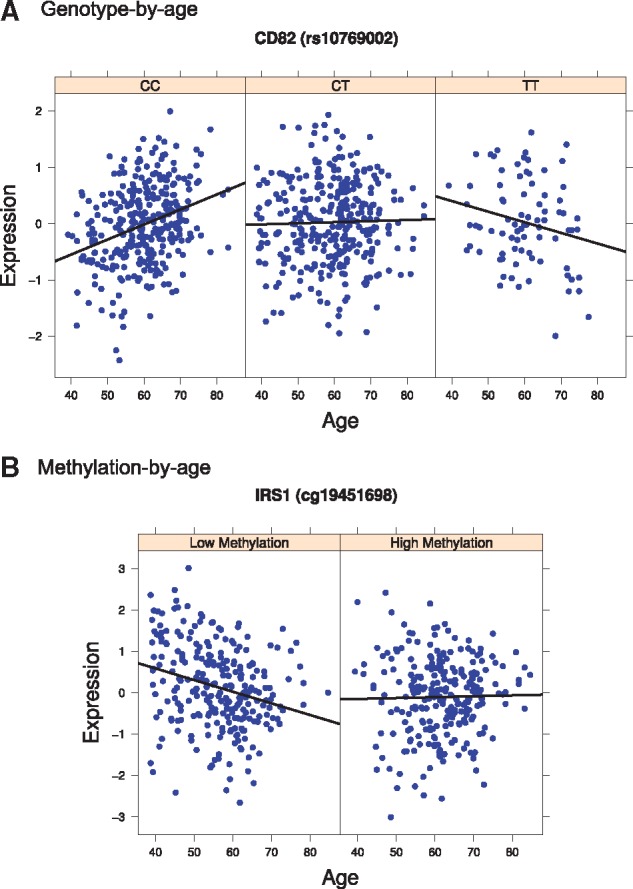
Interacting effects of aging on gene expression: The two plots show the effects of genotypes (eQTL) and methylation on gene expression can be modulated by age. (**A**) The graph shows a genotype-by-age expression quantitative trait locus (GxAge-eQTL) in fat tissue affecting the expression of the *CD82* gene. The expression of the reported exon increased with age in homozygous individuals for the CC alleles in rs10769002. Homozygous individuals for the alternative allele (TT) showed a decreased in expression with age. (**B**) The graph shows a methylation-by-age interaction affecting gene expression. The expression of the *IRS1* gene decreased with age in individuals with the methylation cg19451698 hypomethylated.

We were able to identify genes with a significant change in variance in gene expression due to environmental factors linked to aging and similar in MZ twins. However, changes in variance of expression can also be the results of changes in genetic regulation (6,16,17). Such effects can be identified as genotype-by-age interactions affecting expression (GxAge). However, it is well known that the power to discover such interaction effects is much reduced compared with standard main effects; for this reason, it is common to restrict the search space to those with known main effects, either genetic or on aging, or by looking for variance/discordance effects (15,18). We used the latter approach and tested *cis*-GxAge regulatory interaction effects for the 12 830 exons which were either 1) differentially expressed with age; 2) showed variance changes with age or 3) discordant in expression between MZ co-twins with age in fat or skin. After multiple testing corrections, we identified one significant GxAge-eQTL, affecting the expression of *CD82* among the genes differentially expressed with age in fat (Fig. 3). We also detected three GxAge-eQTLs among the genes that were discordant between MZ co-twins for expression in skin, affecting expression of *CNKSR1*, *ACO1* and *ACSS2* (Supplementary Material, Fig. S9 and Files S7–S9). Despite the inherent challenges in identifying interaction effects, we here identify four GxAge effects on gene expression with a relatively modest sample size.

Given we identified genes with age-dependent changes in variance of expression, but few changes in genetic regulation which could explain them, we also explored environmentally driven changes that may accumulate with age in different manners among individuals and could explain some of the age-related changes in expression. For this, we chose to explore the effects of age in methylation, since methylation levels and discordance in methylation between MZ twins has been shown to accumulate globally with age in promotor regions (19,20). Following the same logic as with genetic effects, we looked for interactions between methylation and age (methylation-by-age) affecting gene expression that would explain the changes in variance observed. For this, we used previously published methylation data collected from the same fat biopsies as our RNA-seq data (21). We focused on genes for which we had observed an association between variance in expression and age with a significance threshold of *P <* 0.1 and investigated a total of 9 genes. We identified a Bonferonni significant methylation-by-age interaction effect on expression of *IRS1* at three methylation probes, the most significant being at probe *cg19451698* (*P* value = 6.6e-05, Fig. 3, Supplementary Material, File S10). This significant interaction implies that the expression of the *IRS1* gene decreases with age in individuals with *cg19451698* hypomethylated. Such an effect was not present in individuals with high levels of methylation in the same region. Our results indicate that environmentally driven changes in genetic regulation are a plausible explanation for age-dependent changes in variance, although their genome-wide relevance may not be large.

### Age effects in expression are shared across tissues

**Figure 4.**
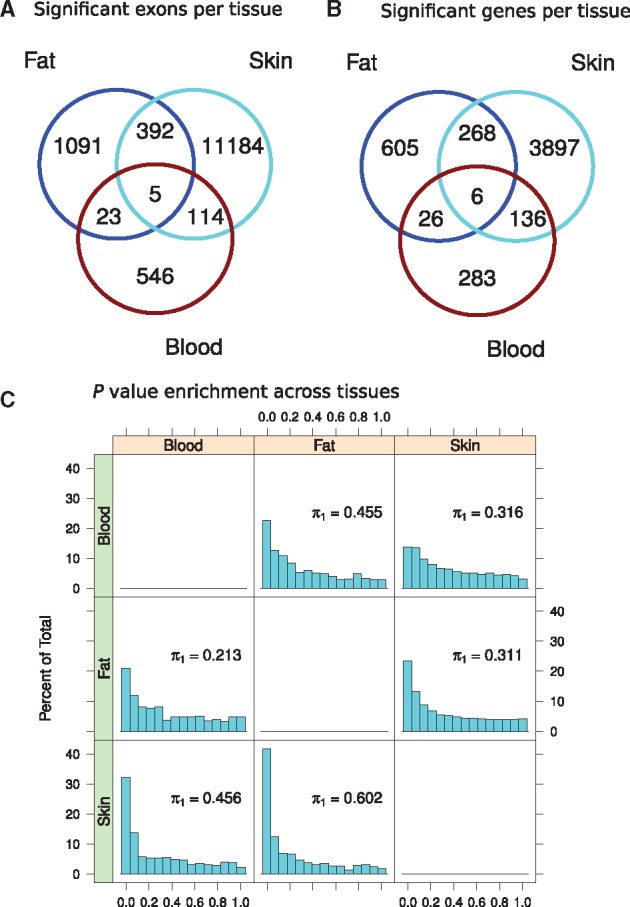
Tissue shared and specific effects of aging in gene expression changes with age. The top venn diagrams show (**A**) the number of exons (left) and (**B**) number of genes (right) significantly associated with chronological age in fat, skin and whole blood. Five exons were commonly associated to age in the three tissues. LCLs were not included, as only 7 exons were significantly associated with age. (**C**) The *P* values of significant exons associated with age in one tissue were extracted from the analysis in the other tissues for enrichment analysis (π1). The histograms show the *P* values for association between expression and age in one tissue (left, green color) if the exons were significantly associated exons in another tissue (top, orange color). As shown in the graphs, age-related signals detected in fat shared an estimated 60.2% of the age effect signal skin tissue and 45.6% with blood.

Finally, we wanted to evaluate how much of the age-related effects on gene expression were shared across all the tissues tested, since previous studies performed in multiple tissues identified a limited number of shared genes associated with age across tissues (3,8,22). Here, of the 5631 genes (36.67%) affected by age in at least one tissue, we were only able to identify five genes significantly associated with age in all the three primary tissues (Fig. 4, Supplementary Material, Fig. S10, Table S6). By pair-wise comparisons between tissues we found that 274 was the largest number of genes significantly associated between two tissues (Fig. 4B). However, this degree of overlap of associated exons across tissues was significant (*P* value < 1e-216, Fishers test) indicating the presence of a common signature of aging across tissues. Furthermore, defining tissue-shared effects based on strict thresholds will underestimate the true sharing between tissues, particularly in blood, which had reduced power to detect associations due to smaller sample size. Enrichment analysis revealed that pairs of tissues shared between 21% (skin and blood) and 60% (fat and skin) of age-related effects in common (Fig. 4) (3). Our results indicate that global biomarkers of aging with effects across multiple tissues are prevalent, but also that there is a strong tissue-specific component to ageing, even between highly related tissues such as fat and skin. This is supported by the findings of multi-tissues studies like GTEx pilot study, which identified 1000s of genes using 9 tissues (22).

## Discussion

In this study, we have explored a large multi-tissue expression dataset to investigate the influence of aging on genetic regulation. We find an increased number of genes differentially expressed with age in line with the larger sample sizes in our study. Furthermore, we have extensively explored the relationship between changes in phenotypic variance with age, an under-studied effect of aging reported in multiple studies and in which global changes in phenotypic variance increase with age (2). This increase in phenotypic variance has been mainly considered a manifestation of a loss in regulatory capacities in aging organisms, and it is often proposed as a link between aging and diseases (10,11). However, our analysis mainly identified individual genes with a decreased variance, contradicting the expectation of a stochastic increase of the phenotypic variance with age due to reduced regulatory capabilities.

Three factors may induce changes in phenotypic mean or variance: genetic variation, environmental variation or an interaction between the two. We were able to investigate these factors due to the unique characteristic of twin data, where genetic and environmental components of variance can be explored. We have successfully used this strategy previously to classify genetic determinants of phenotypic variance in gene expression (23) and GxE interactions affecting allelic specific expression (24). Others have also explored the differences in gene expression between genetically identical twins (MZ) with age (25), but the possible causes of these changes in variance have not been fully explored before. Besides identifying genes with an changed in variance with age, we observed that genes with expression affected by age were highly heritable (Fig. 2, Supplementary Material, File S4) suggesting, as previously reported, that age modulates genetic regulation of expression. Genes and pathways associated with longevity and age-related changes are often genetically regulated in older organisms with low levels of stochasticity and higher levels of heritability (6,11,26). Therefore, genes affected by age and highly heritable in older individuals are longevity candidate genes than may increase our understanding of the relationship between longevity and healthy aging.

We attempted to identify genetic and environmental factors involved in the changes of variance with age by testing for GxE interactions. We were able to identify a significant GxAge-eQTL in fat tissue acting on the gene *CD82* (*rs10769002*). This gene is associated with tumor progression as it codes for a metastasis suppressor glycoprotein highly correlated with *TP53 (p53)* and increase in its expression has been associated with overall better survival to cancer (27). In our analysis, we observed that individuals homozygous for the reference allele increased gene expression with age compared with the alternative allele. Therefore, it is possible that the alternate allele in *rs10769002* may be a risk factor for some types of cancer in older individuals. Three other examples were identified in skin tissue for genes also previously implicated in cancer and metabolism. Our search for environmentally induced changes in gene expression regulation with age identified *IRS1* as a gene which expression changes as a consequence of an age-methylation interaction. The *IRS1* gene has been associated with T2D, an age-related disease, and it has also been found to have T2D associated DMRs nearby (28). In conclusion, we identify changes in phenotypic variance with age that would be explained by GxE and changes in regulation, suggesting that damage accumulation is not the only explanation to the observed change in phenotypic variance with age for whole organism traits phenotypes. Moreover, we show that the study of phenotypic variance with age in gene expression manifesting in the form of GxE interactions may identify new candidate genes relevant for longevity and age-related diseases.

**Figure 5.**
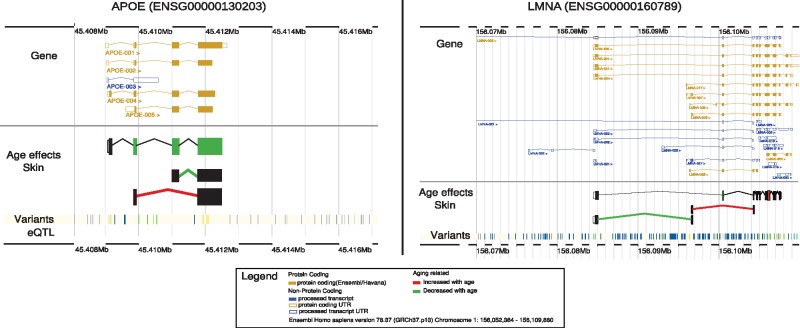
Age effect for *APOE* and *LMNA*. On the left, a scheme of the *APOE* gene and the multiple protein coding transcripts variants (yellow) and non-coding processed transcripts (blue) produced by the gene. In the skin tissue, three exons and one link decreased their expression with age (green-coloured exon and link between exons); and one link increase its expression (red-coloured link). On the right, a scheme of the *LMNA* gene and the age affects in the skin tissue. Two exons were affected in their expression by age by increasing expression (red coloured exon, corrected *P*-val < 0.1) and decrease expression with age (green-coloured exon). Furthermore, two links were significantly associated with age in their expression (corrected *P* val < 0.05). Our results suggested an increase in the production of isoforms using alternative 5’.

Many of the genes highlighted throughout this work have been previously associated with age-related diseases. The associations between aging and disease and genetics and disease has been extensively catalogued by epidemiological and GWAS studies, respectively (2,29), but the association between specific genes with a disease in the context of the aging process remains elusive. Our analyses have identified thousands of genes changing their expression due to genetic or environmental factors with age, some of which may explain the influence of aging in the onset and outcome of diseases. From the genes reported to show age effects in expression, we chose to highlight two examples of genes linked to age-related disease to illustrate the multiple changes that aging may induce in any given gene. The first example is the *APOE* gene, a gene which expression has been associated with Alzheimer and cardiovascular diseases and with genetic variants at the *TOMM40/APOE/APOC1* locus near the gene also associated with longevity (30). Our analysis showed that the expression of multiple exons and links of *APOE* change with age in skin tissue, producing different isoforms that can potentially induce changes in the activity of the gene (Fig. 5). Our GxAge-eQTL analysis reported a nominally significant *P* value of 0.014. Given the strong association between expression and disease, such an effect could modulate age-related development and progression of disease. The second example we chose to highlight here involves the *LMNA* gene (Fig. 5), which is causal of the Hutchinson-Gilford progeria syndrome. This syndrome is characterized by accelerated aging features as a consequence of the accumulation of a truncate progerin isoform of *LMNA.* The progerin transcript increases with age in normal cells (31), with its protein known to accumulate in human skin in an age-dependent manner (32). We reported changes in expression of exons (adjusted *P* values < 0.1) and links (adjusted *P* values < 0.05) between exons consistent with the production of different alternative isoforms in an age-dependent manner. Furthermore, an eQTL affecting the expression of the gene is active in skin, blood and LCLs tissues. The peak LCL eQTL (*rs915179*) has been previously linked to exceptional longevity in humans (33,34). These examples illustrate that studying the global effects of the aging process may lead to the identification of gene involved in age-related diseases.

In summary, by performing a large human transcriptomic study of aging in multiple tissues, we found that the shared effect of aging in humans across four tissues as well as the number of affected genes is larger than previously reported. We also report that the global effect of age in gene expression is small (median variance explained by age is between 2.2 and 5.74%) compared with the genetic effects or other environmental effects. When compared with the global effect of genetic factors on gene expression, and the success of eQTLs studies on finding genetic regulatory elements, the low age-related values may explain the difficulties in identifying biomarkers of aging with gene expression, and highlight the need of larger sample sizes that account for genetic variation. Moreover, we have shown that age alters gene expression in multiple complex ways, including variance, mRNA maturation and genetic regulation. Many of these affected genes have been linked to age-related diseases, showing the need for future studies into the relationship between age-related changes in gene expression and its regulation, and age-related diseases. This is particularly relevant for genome wide association studies (GWAS) where eQTL are routinely used to identify target genes of genetic associations without accounting for the effects of age.

## Materials and Methods

### Study design

Sample collection, and mRNA extraction have been described in detail in (21). In sort, 856 Caucasian female individuals (336 MZ and 520 DZ twins) from the TwinsUK Adult twin registry (35) were recruited with a ranged age from 39 to 85 years (mean 59 years). Tissue and blood samples were extracted on the same visit per pair of twins, matching in this way their age at collection. Samples were prepared for sequencing and processed as described in (23) and (24). The number of monozygotic (MZ), dizygotic (DZ) and unrelated individuals (individuals with no relatives in the dataset) included in the final analysis per tissue are described in Supplementary Material, Table S1.

### Exons and links quantification

The 49-bp sequenced paired-end reads were mapped to the GRCh37 reference genome [The International Human Genome Sequencing Consortium, (36)] with BWA v0.5.9 (37). We use genes defined as protein coding in the GENCODE v10 annotation (38), removing genes with more than 10% zero read count in each tissue. For the analysis presented in this paper, only exons from protein-coding genes and LincRNAs from verified loci (level 1) and manually annotated (level 2) were investigated. We calculated the relative quantification of splicing events using Altrans (14). Read counts assigned to links and exons were scaled to 10 million reads.

Supplementary Material, Table S7 shows the total number of exons and genes sequenced per tissue, as well as the total number of exons, genes used in the analysis here presented.

### Genotying and imputation

Genotyping of the TwinsUK dataset (*N =* ∼6000) was done with a combination of Illumina arrays as described in (21,23,24). Samples were imputed into the 1000 Genomes Phase 1 reference panel (data freeze, 10/11/2010) (39) using IMPUTE2 (40) and filtered (MAF < 0.01, IMPUTE info value < 0.8).

### Splicing junction quantifications

We calculated the relative quantification of splicing events using Altrans (14). The method makes use of mate pairs mapped to different exons to count “links” between two exons based on the GENCODE v10 annotation for level 1 and 2 from protein coding genes and lincRNA. Exons that overlap were grouped into “exon groups” to identify unique portions of each exon from an exon group. The unique portions were used to assign reads to an exon. The quantitative metric produced by Altrans is the fraction of one link's coverage over the sum of overages of all the links that the primary exon produced. The values range from 0 to 1, representing the proportion of a give link among all the links produced by the primary exon. The metric is calculated in 5'-to-3' (forward) and 3'-to-5' (reverse) directions to capture splice acceptor and donor effects respectively. Supplementary Material, Table S8 shows the total number of links identify per tissue, as well as the total number of links per gene detected.

### Age effects on mean exon expression and links

Rank normalized reads per exon or links were used to assess the age effect on exon expression mean. A linear mixed model was fitted to examine age effect on gene expression in R (41) with the lmer function in the lme4 package (42). Confounding factors in all models included fixed (primer insert size, GC content mean and, only for blood samples, batch) and random effects (primer index, date of sequencing, family relationship and zygosity). The *P* values to assess significance for age effect were calculated from the Chi-square distribution with 1 degree of freedom using likelihood ratio of nested models as the test statistic. A set of 100 permutations were used to adjust for multiple testing. Expression values were permuted while maintaining samples from twin pairs together. To correct for the number of exons per genes, which would allow genes with more exons to have more significant associations by chance than genes with fewer exons, we calculated adjusted *P* values in 16 groups, one per group of genes with similar number of exons. The adjusted *P* values were calculated as the proportion of permuted statistics more significant, divided by 100. Adjusted *P* values < 0.05 were considered significant. A gene was considered as significantly affected by age in its expression if at least one exon was significantly associated with it.

### Tissue shared effects

For each pair of tissues comparison we extracted *P* values of exons in one tissue (e.g. skin) from significantly age associated exons in other tissue (e.g. fat). The *P* values distributions were used to assess the enrichment of age associated exons in other tissues. Analysis were performed in largeQvalue (43), an implementation of the R statistical software qvalue package (44), for large datasets.

### Age effect on variance of gene expression

Residuals removing technical covariates and family structure were used to assess the association for variance and age per tissue. Residuals were extracted from a linear mixed model fitted with the lmer function in the lme4 package (42) using R. Confounding factors in all models included fixed and random effects as detailed above. The residuals were fit on a LOESS function including age as response variable. Residuals from the LOESS regression were squared root to give a measure of the distance from the mean expression with age. A Spearman correlation test between this ‘distance’ and the age was used to assess evidence for an age effect on variance.

### Age effect on discordance of gene expression

Residuals removing only technical covariates were used to assess the change in discordance of gene expression with age per tissue from complete MZ pairs of twins (Supplementary Material, Table S1). Association with age was assessed by regressing the maximum expression of each twin pair on the expression of the sibling plus age to detect whether the relationship between maximum and minimum expression was conditional on age. Multiple testing was assess using 100 permutations and as described for the expression association.

### Fat methylation analysis

Infinium HumanMethylation450 BeadChip (Illumina Inc, San Diego, CA) was used to measure DNA methylation. Details of experimental approaches have been previously described (45). To correct the technical issues caused by the two Illumina probe types, the beta mixture quantile dilation (BMIQ) method was performed (46). The methylation data were also background corrected. DNA methylation probes that mapped incorrectly or to multiple locations in the reference sequence were removed. Probes with >1% subjects with detection *P* values > 0.05 were also removed. Subjects with more than 5% missing probes were also removed. All probes with non-missing values were included.

### Effect sizes and heritability analysis

We calculated effect size of age in expression and methylation from the normalized data and as a proportion of variance attributed to age over the total variance in exon expression. We also calculated the variance attributed to additive genetic effects, common environment and unique environment. Variance components were calculated from a linear mixed model, as previously described in (21), and (47) using all available complete twin pairs per tissue (Supplementary Material, Table S1). The model was fitted as described above.

### Genotype-by-age and methylation-by-age interactions

Expression residuals removing technical covariates and family structure were used to assess the association of exons and genetics variance interacting with age. To identify genotype-by-age interactions affecting gene expression, we performed a linear regression of the residuals of each exon on the SNPs in a 1Mb window around the transcription start site for each gene, using a linear model in R. Only SNPs with MAF ≥ 0.05 were tested. We used 10 permutations to assess the significance of the interactions for exons with age-related effects, namely mean expression changes, variance changes and discordant effects. We used a similar strategy as used by (48) and based on (49). A linear model with main effects but without an interaction term was used to extract residuals for each exon-SNP association test. The residuals were permuted (10 times) and used in a linear association with a model for the interacting term (GxAge). *P* values from this analysis were stored and used to adjusted *P* values correcting for the number of exons per genes, as described before.

Methylation-by-age interaction analysis used expression and methylation residuals after removal of technical covariates and accounting for family structure. A linear model was used to test the association between expression and methylation levels with age. Significant associations were considered those with a *P* value < 1.0e-4 (Bonferroni correction).

## Ethics Statement

This project was approved by the ethics committee at St Thomas' Hospital, London, where all the biopsies were carried out. Volunteers gave informed consent and signed an approved consent form prior to the biopsy procedure. Volunteers were supplied with an appropriate detailed information sheet regarding the research project and biopsy procedure by post prior to attending for the biopsy.

## Data Availability

RNAseq data are available from the European Genome-phenome Archive (EGA) under the accession EGAS00001000805. Methylation data were downloaded from ArrayExpress, accession number E-MTAB-1866.

## Supplementary Material

Supplementary Material is available at *HMG* online.

## Supplementary Material

Supplementary Figures

Supplementary File 1

Supplementary File 2
