# Supplementary material for: Age-dependent changes in mean and variance of gene expression across tissues in a twin cohort
Source: Hum Mol Genet. 2017 Dec 8;27(4):732–41. doi: 10.1093/hmg/ddx424 (PMC5886097; doi:10.1093/hmg/ddx424)
Supplement: Supplementary Figures [file supplementalfigures_ddx424.docx]

**Supplementary Figures and Tables**


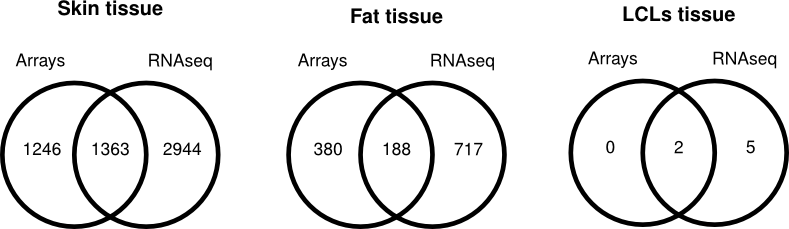


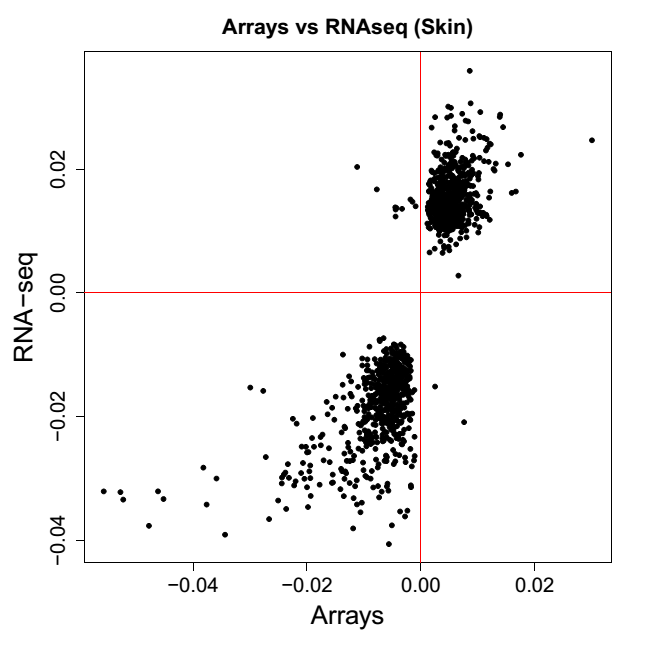

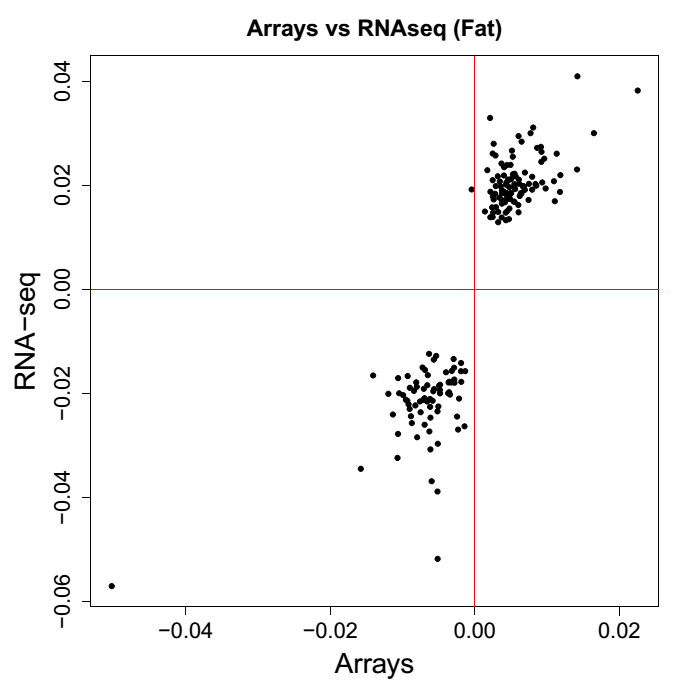


**Supplementary Figure 1 |** Top: Venn Diagrams per tissue comparing differentially expressed genes in array expriments (Glass et al, 2012) and gene swith at least an exon differentially expressed with age from RNA-seq data. Bottom: we compared the direction of effect of significantly affected genes with age in both technologies in skin tissue and fat tissues. The plot shows how RNAseq data replicates the same direction of effect for age in the majority of the significant genes in both technologies, which should be expected as they relate to quantifications of the same underlying phenotype in the same samples. For the very few genes with opposite effects, alternative splicing may explain the differences


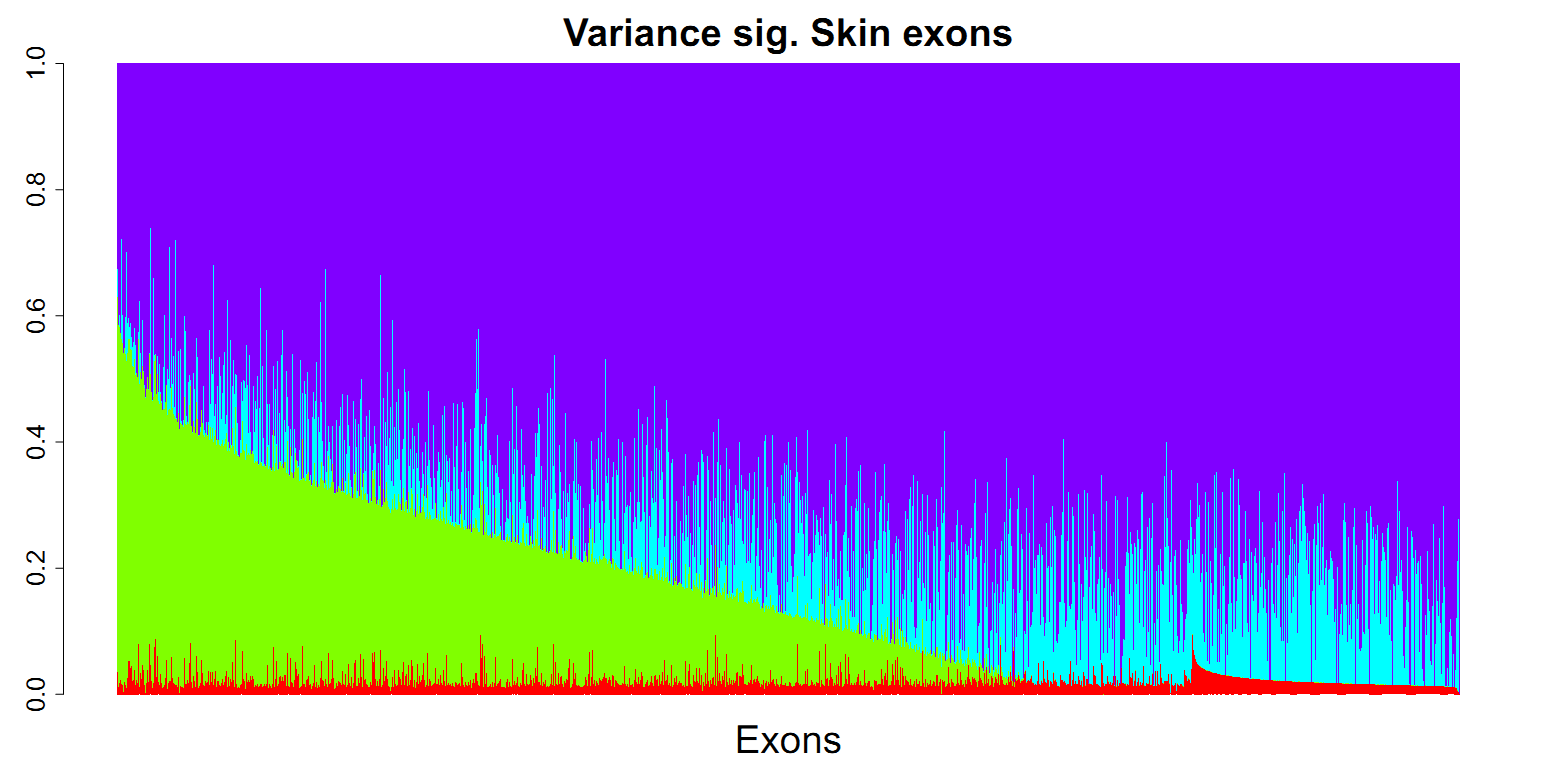


**Supplementary Figure 2 |** Barplot of variance decomposition in 11,695 exons significantly associated to age in skin. Each bar show the proportion of variance attributed to age effect (red); variance attributed to genetics (green); variance attributed to common environment (cyan); and variance attributed to the unique environment (purple). Bar were ordered from higher variance attributed to genetics (left) to lower.


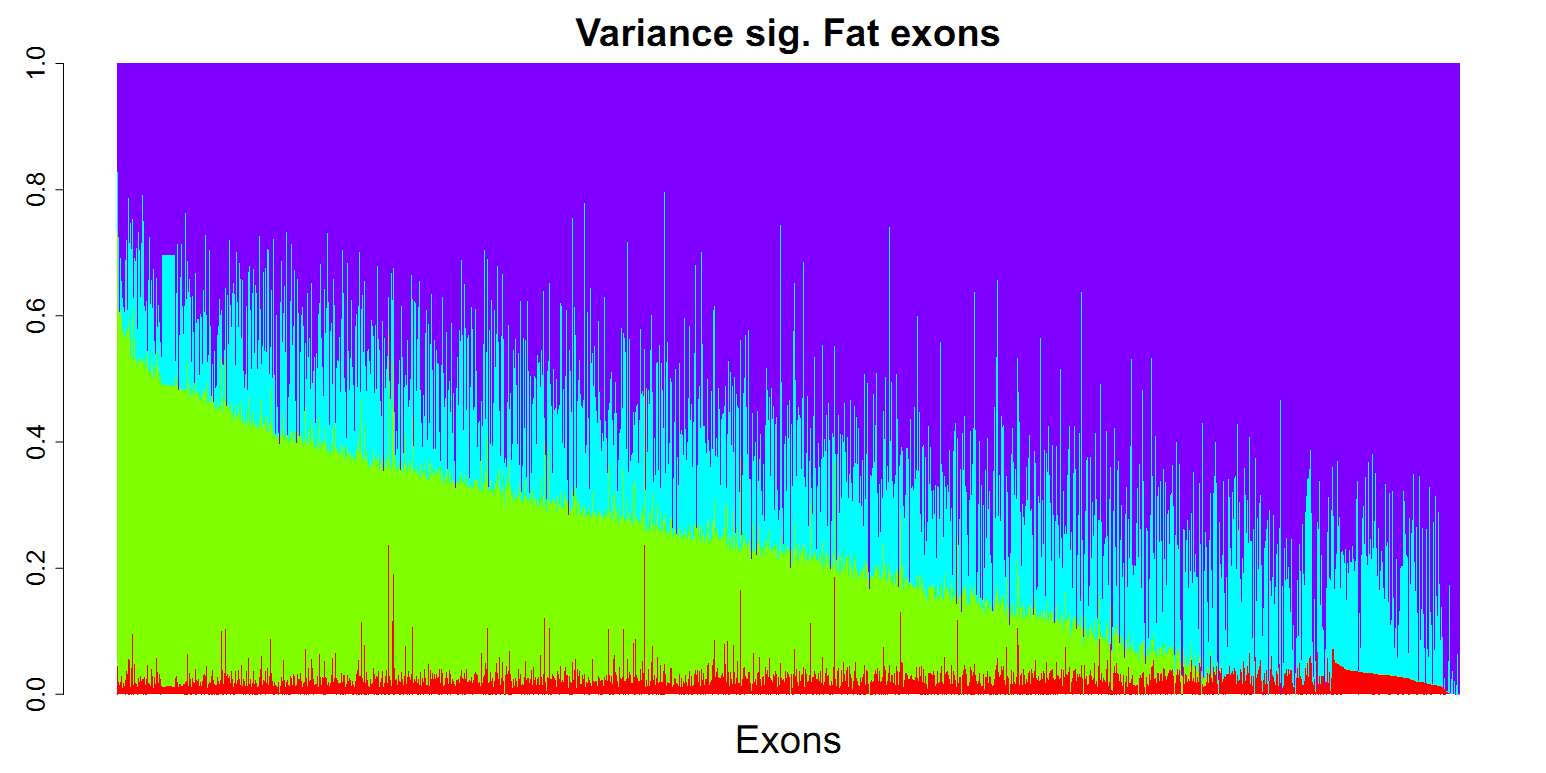


**Supplementary Figure 3 |** Barplot of variance decomposition in 1,511 exons significantly associated to age in fat. Each bar show the proportion of variance attributed to age effect (red); variance attributed to genetics (green); variance attributed to common environment (cyan); and variance attributed to the unique environment (purple).


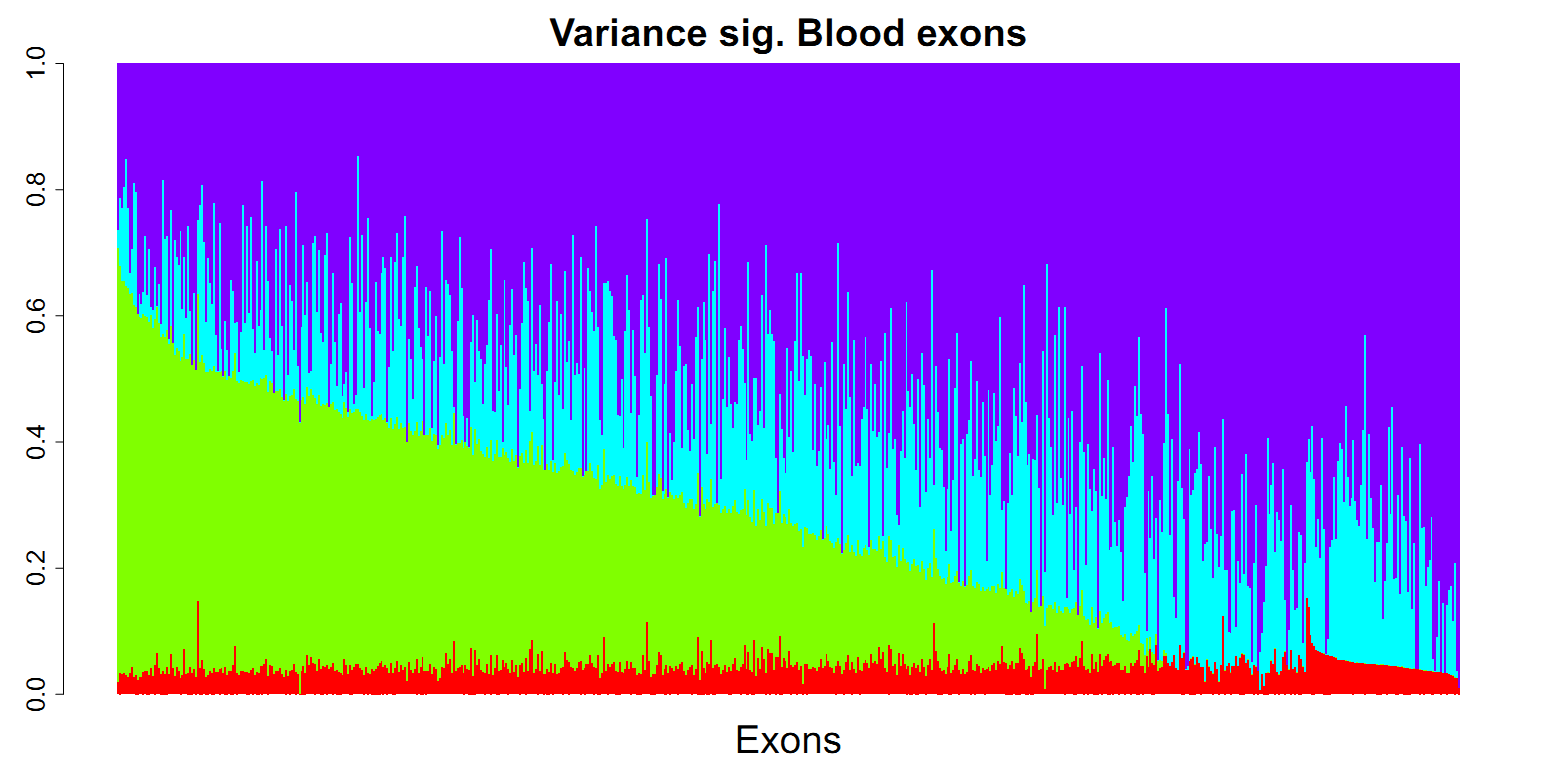


**Supplementary Figure 4 |** Barplot of variance decomposition in 688 exons significantly associated to age in whole blood. Each bar show the proportion of variance attributed to age effect (red); variance attributed to genetics (green); variance attributed to common environment (cyan); and variance attributed to the unique environment (purple).

**
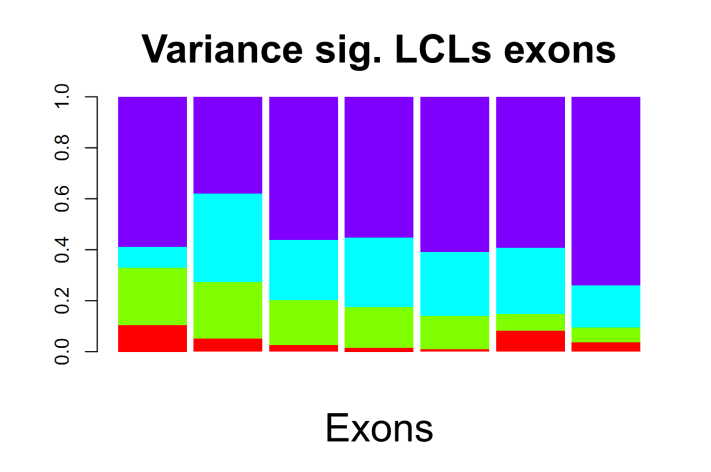
 Supplementary Figure 5 |** Barplot of variance decomposition in 7 exons significantly associated to age in LCLs. Each bar show the proportion of variance attributed to age effect (red); variance attributed to genetics (green); variance attributed to common environment (cyan); and variance attributed to the unique environment (purple).


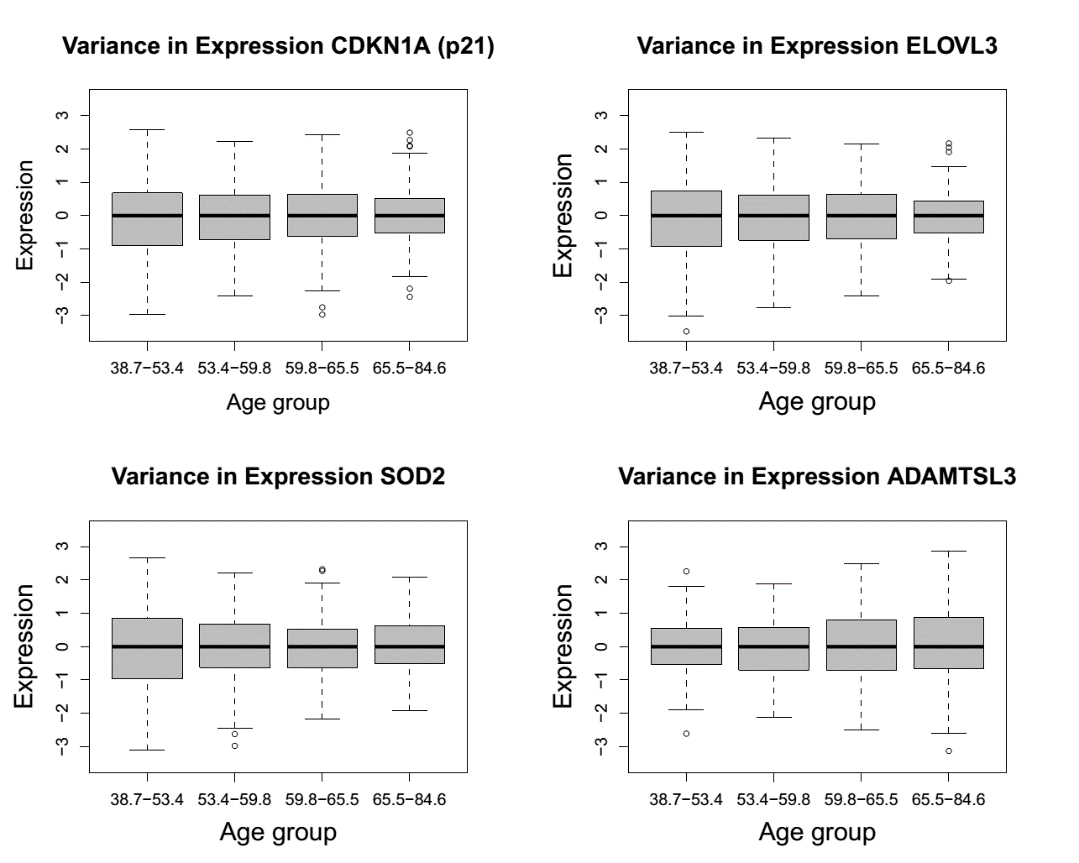


**Supplementary Figure 6|** Examples for significant effect of age in variance of exon expression in skin. For this plot, individuals were grouped by ages, as indicated on the x-axis, with their expression values for the genes centered by the median expression, showing a decrease (top and bottom left plots) and an increased (bottom right) in variance with age.


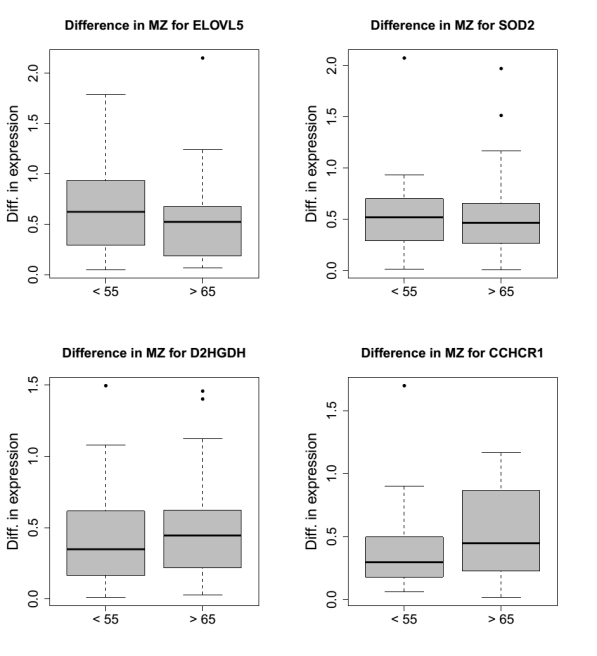


**Supplementary Figure 7 |** Barplots showing the difference in expression between MZ twins younger than 55 years old and older than 65 years old for genes with a significant decrease (top plots) and increase (bottom plots) in variance.


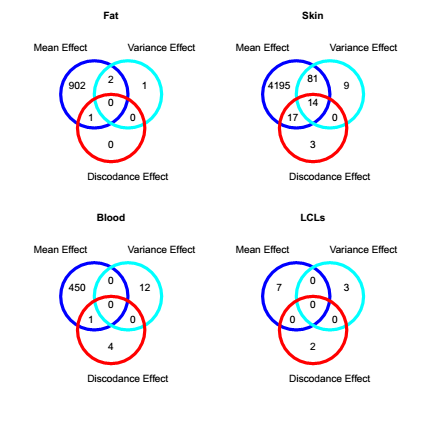


**Supplementary Figure 8 |** Venn diagram showing the overlap in genes with at least one exon affected by changes in the mean (DE genes), variance and differences between MZ twins (discordance) in each tissue.


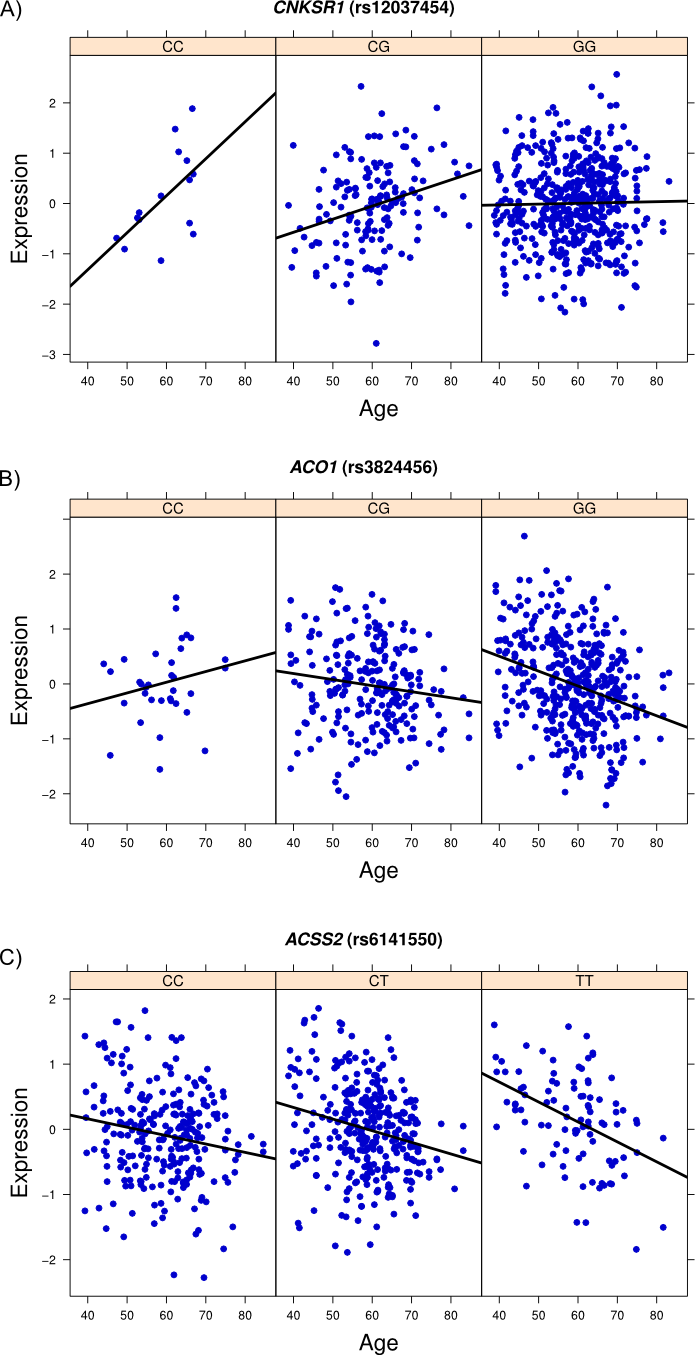
 **Supplementary Figure 9 | Interacting effects of aging on gene expression:** All the graphs show a genotype-by-age expression quantitative trait locus (gxa-eQTL) in skin tissue affecting the expression of three genes.


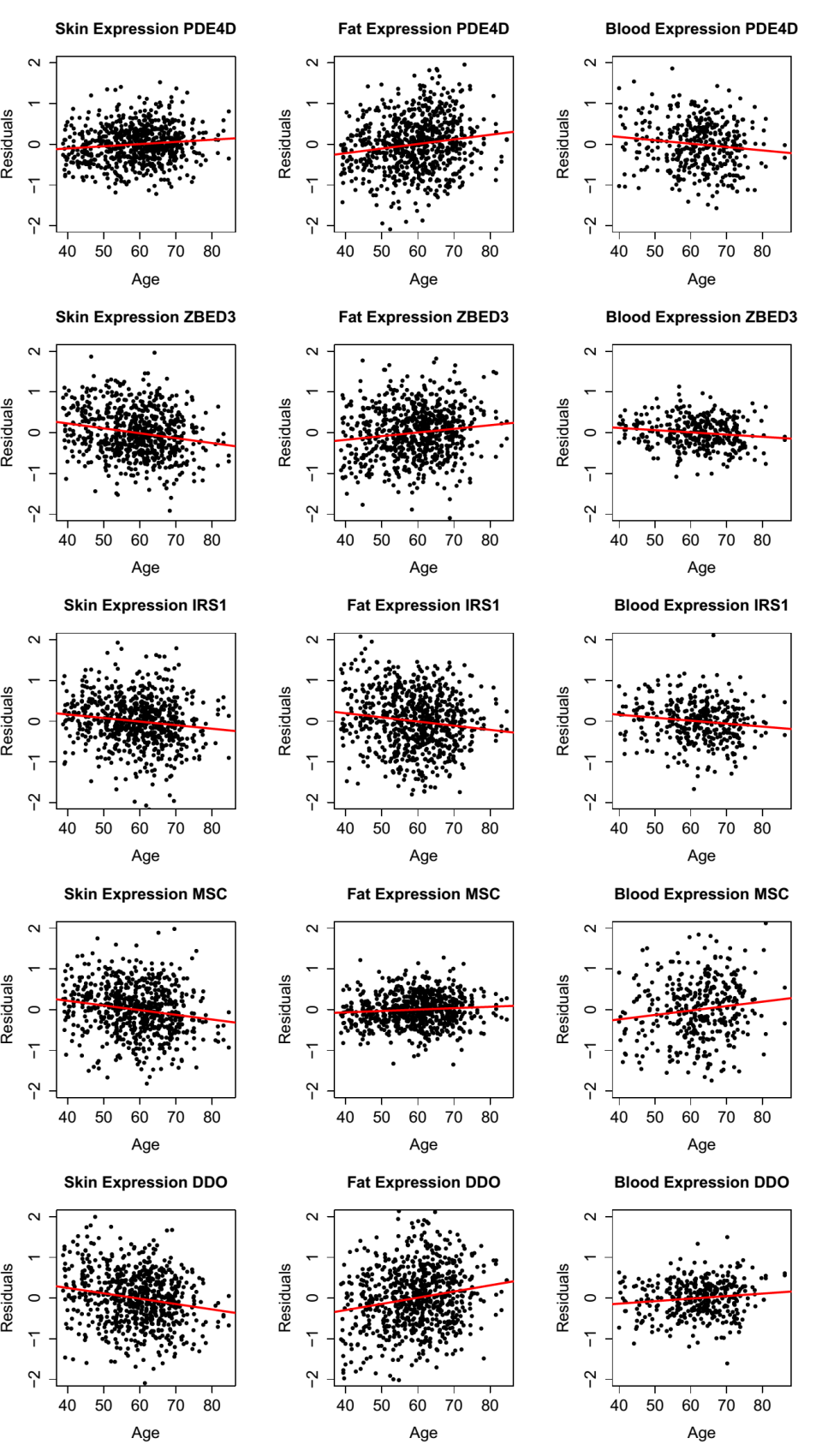


**Supplementary Figure 10 |** Genes commonly affected by age in the three primary tissues: skin, fat and whole blood. The graph shows the residuals from a linear mixed model removing technical covariates and family structure. The red lines are linear models fitted with the residuals association with age, indicating therefore the direction of effect of age in the expression of the exons plotted.

Tables

|  | **Fat** | **Skin** | **LCLs** | **Whole Blood** |
| --- | --- | --- | --- | --- |
| **Samples** | 766 | 716 | 814 | 384 |
| **MZ pairs** | 131 | 114 | 137 | 69 |
| **DZ pairs** | 187 | 173 | 217 | 91 |
| **Unrelated** | 130 | 142 | 106 | 64 |

**Supplementary Table 1 |** Number of monozyigous (MZ), dizygous (DZ) and unrelated individuals (individuals with no relatives in the dataset) included in the final analysis per tissue are described on the following.

|  | **Fat** | **Skin** | **Whole Blood** | **LCLs** |
| --- | --- | --- | --- | --- |
| **Exons** | 1,511 | 11,695 | 688 | 7 |
| **Increased expression** | 840 (55.6%) | 7,094 (60.6%) | 356 (51.7%) | 4 (57.1%) |
| **Decreased expression** | 671 (44.4%) | 4,601 (39.4%) | 332 (48.2%) | 3 (42.8%) |
| **Protein coding** | 1,451 | 11,528 | 680 | 7 |
| **LincRNA** | 60 | 167 | 8 | 0 |
| **Genes** | 905 | 4,307 | 451 | 7 |
| **Mean sign. exons per gene** | 1.67 | 2.71 | 1.52 | 1 |
| **Genes with 1 sign. exon** | 685 (75.69%) | 2,333  (54.16 %) | 347 (76.94%) | 7  (100%) |
| **Genes with all exons sig.** | 366  (40.44%) | 904  (20.98%) | 148 (32.81%) | 2  (28.57%) |

**Supplementary Table 2 |** Number of exons and genes significantly associated with age in all tissues. The numbers of those genes that are protein coding genes and LincRNAs are also indicated. The last three rows show the average number of exons per significant gene, number of genes with only exons significantly associated with age and number of genes with all the exons significantly associated with age, respectively. The percentage of the age associated genes are show under each number.

|  | **Fat** | | | | **Skin** | | | | **Blood** | | | | **LCLs** | | | |
| --- | --- | --- | --- | --- | --- | --- | --- | --- | --- | --- | --- | --- | --- | --- | --- | --- |
|  | **All** | **DE** | **Variance** | **Discordance** | **All** | **Age** | **Variance** | **Discordance** | **All** | **Age** | **Variance** | **Discordance** | **All** | **Age** | **Variance** | **Discordance** |
| **Age** | 0.0012 | 0.0287 | 0.0601 | 0.0263 | 0.0026 | 0.0224 | 0.0822 | 0.0658 | 0.0039 | 0.0542 | 0.0035 | 0.0225 | 0.0006 | 0.0366 | 0.0030 | 0.0018 |
| **Genetics** | 0.0809 | 0.2228 | 8.9e-14 | 0.2649 | 0.0856 | 0.1275 | 0.3513 | 0.2380 | 0.1301 | 0.2333 | 0.2037 | 0.1560 | 0.1089 | 0.2032 | 0.1070 | 0.0901 |
| **Commo Env.** | 0.0236 | 0.0573 | 0.172 | -0.1066 | 0.0000 | 0.0162 | -0.1017 | -0.066 | 0.0380 | 0.0341 | 0.0716 | 0.0293 | 0.0971 | 0.1312 | 0.1966 | 0.0599 |
| **Unique Env.** | 0.8550 | 0.6655 | 0.690 | 0.8094 | 0.8566 | 0.7662 | 0.6778 | 0.7512 | 0.7403 | 0.6214 | 0.6055 | 0.7179 | 0.7320 | 0.6214 | 0.6224 | 0.7644 |
| **N. of exons** | 101,133 | 1,511 | 3 | 1 | 96,736 | 11,695 | 239 | 40 | 71,393 | 688 | 13 | 5 | 98,372 | 7 | 3 | 2 |

**Supplementary Table 3 |** Summary of mean proportion of variance attributed to age, genetics, common environment and unique environment, for exons affected by age in their variance (Variance) and exons discordant for expression with age (Discordant). The last row indicates the number of exons significant for each category (corrected *P*value < 0.05). ). In general, age explained a small proportion of the variance attributed to gene expression. However, for exons affected by age in their expression, the genetic component (heritability) explained significantly higher proportion of the variance in expression compare to the rest of the genes in fat, skin and blood tissues (willconox test Pvalue < 2.1e-17).

| Tissue | Mean Heritability Young | Mean Heritability  Old | Mean Variance exp. Age Young | Mean Variance exp. Age Old | Unique Environment  Young | Unique Environment Old |
| --- | --- | --- | --- | --- | --- | --- |
| Skin | 0.21 | 0.14 | 0.005 | 0.003 | 0.80 | 0.83 |
| Fat | 0.12 | 0.20 | 0.008 | 0.003 | 0.84 | 0.78 |
| Blood | 0.20 | 0.28 | 0.007 | 0.009 | 0.70 | 0.67 |
| LCLs | 0.19 | 0.16 | 0.003 | 0.001 | 0.67 | 0.73 |

| Tissue (mean age) | Pairs of young Twins | Pairs of old Twins |
| --- | --- | --- |
| Skin (58.8) | 135 | 152 |
| Fat (59.3) | 150 | 168 |
| Blood (61.8) | 73 | 87 |
| LCLs (59.1) | 165 | 189 |

**Supplementary Table 5 |** Top table: Analysis of variances explained by age and genetics between young pair of twins and old pair of twins. Bottom table: Pair of twins used for each of the analysis presented in the top table.

| **Exon** | **GeneName** | **Chr** | **TSS** | **P value**  **Fat** | **Beta Age**  **Fat** | **P value**  **Skin** | **Beta Age**  **Skin** | **P value**  **Blood** | **Beta Age**  **Blood** |
| --- | --- | --- | --- | --- | --- | --- | --- | --- | --- |
| ENSG00000113448.11_58264865_58270907 | PDE4D | chr5 | 59284544 | 2.15E-07 | 0.02078 | 0.00103 | 0.00966 | 0.00074 | -0.01471 |
| ENSG00000132846.5_76367897_76373720 | ZBED3 | chr5 | 76375207 | 2.49E-05 | 0.01769 | 3.22E-09 | -0.02289 | 0.00166 | -0.01710 |
| ENSG00000169047.4_227659705_227664475 | IRS1 | chr2 | 227657101 | 1.09E-05 | -0.01876 | 0.00015 | -0.01187 | 0.00176 | -0.01918 |
| ENSG00000178860.8_72753784_72754982 | MSC | chr8 | 72754982 | 0.00113 | 0.01467 | 1.59E-08 | -0.02374 | 0.00018 | 0.02339 |
| ENSG00000203797.4_110712974_110714545 | DDO | chr6 | 110713980 | 1.54E-08 | 0.02357 | 3.72E-08 | -0.02299 | 0.00165 | 0.01926 |

**Supplementary Table 6 |**List of significant exons differentially expressed with age in all tissues.

|  | **Fat** | **Skin** | **LCLs** | **Whole Blood** |
| --- | --- | --- | --- | --- |
| **Exons** | 118,643 | 114,376 | 116,528 | 85,811 |
| **Genes** | 19,111 | 19,900 | 18,230 | 16,148 |
| **Tested exons** | 101,133 | 96,736 | 98,372 | 71,393 |
| **Tested Genes** | 13,493 | 14,240 | 12,098 | 11,433 |
| **Mean exons per gene** | 7.49 | 6.79 | 8.13 | 6.24 |

**Supplementary Table 7 |** Total number of exons and genes sequenced per tissue, as well as the total number of exons, genes used in the analysis here presented. We use genes defined as protein coding in the GENCODE v10 annotation removing genes with more than 10% zero read count in each tissue. For the analysis presented in this paper, only exons from protein coding genes and LincRNAs from verified loci (level 1) and manually annotated (level 2) were investigated.

|  | **Fat** | **Skin** | **LCLs** | **Whole Blood** |
| --- | --- | --- | --- | --- |
| **Links** | 221,057 | 179,012 | 259,903 | 184,105 |
| **Genes** | 4,572 | 4,289 | 5,502 | 5,185 |
| **Tested links** | 179,675 | 146,908 | 227,594 | 86,777 |
| **Tested Genes** | 3,876 | 3,557 | 4,673 | 1,590 |

**Supplementary Table 8 |** Total number of links identify per tissue, as well as the total number of links per gene detected is shown in the following table. Those link belong to genes included in table 3.
