# Supplementary material for: Age-dependent changes in mean and variance of gene expression across tissues in a twin cohort
Source: Hum Mol Genet. 2017 Dec 8;27(4):732–41. doi: 10.1093/hmg/ddx424 (PMC5886097; doi:10.1093/hmg/ddx424)
Supplement: Supplementary File 2 [file supplementary_file11_ddx424.pdf]

```
# Copyright statement comment Author comment File description comment, including
# purpose of program, inputs, and outputs source() and library() statements

# Scripts used in analyses and plots presented in Vinuela et al, 2016
# For simplity while reading the paper, the analysis are here presented in the
# same order than in the paper.

# Index
# 1) Calculate age effect on gene expression
# 2) Calculate age effect on splicing of gene expression
# 3) Calculate age effect on variance of gene expression
# 4) Calculate age effect on discodance of gene expression in MZ twins
# 5) Calculate genotype-by-age interactions
# 6) Calculate methylation association with age and expression

# 1.1) Script to calculate age effect on gene expression in RNAseq data deposited
in EGA
# under the accession EGAS00001000805.

# Run the models.  This function loops over the exon reads files and run the
# association between expression and age in model Fm1. The model Fm2 is the null
# model, without the variable of interest.  Pvalues are claculated from the anova
# test comparing both linear mixed models.  cat prints the results that the sink
# function will output.


RunModelsAge <- function(reads, age, insertSizeMode, gcMean, primerIndex, date,
zygosity,
        family, nIds) {
    # Computes the association between expression and age using linear mixed models
    # Args:
    #  reads: data.frame with read counts per exon to be tested
    #  family: factor variable identifying families. This is coded with the family
ID (use same ID
    #    for siblings)
    #  zygosity: factor variable identiying zigosity of individuals (MZ
    #    or DZ). Same number for MZ twins (family ID), different number for DZ
twins
    #  insertSizeMode: numeric variable describing insert size properties of the
    #    mRNAsequence
    #  gcMean: numeric variable describing mean values of GC content per
    #   sample
    #  primerIndex: factor variable describing primer index used for sequencing
    #  date: factor variable identifying samples sequenced the same date (a.k.a.
batch
    #    effect)
    #  age: numeric variable for the age of the individuals at the time of
    #   sample extraction
    #  n_ids: numeric variable with the number of individuals
    # Returns:
    #  A full text line with the fillowing columns: exonID, ChiSquare for
    # anova test, Pvalue for anova test, fix effect (beta) for age, fix effect
(beta)
    # for insertSizeMode, fix effect (beta) for gcMean.

    require(lme4)
    require(Matrix)

    nReads <- dim(reads)[1]
    readsId <- NULL
    iReads <- NULL

    for (iReads in 1:nReads) {
        readsId <- reads[iReads, c(1)]
        yIn <- as.matrix(as.double(reads[iReads, 5:nIds]))
```

```
        Fm1 = Fm2 = NULL

        ######################################################################
        Fm1 <- lmer(yIn ~ 1 + age + insertSizeMode + gcMean + (1 | primerIndex) +
            (1 | date) + (1 | zygosity) + (1 | family), REML = FALSE)
        Fm2 <- lmer(yIn ~ 1 + insertSizeMode + gcMean + (1 | primerIndex) + (1 |
            date) + (1 | zygosity) + (1 | family), REML = FALSE)
        ######################################################################

        cat(paste(as.character(reads[iReads, 1]), anova(Fm1, Fm2)$Chis[2], anova
(Fm1,
            Fm2)$Pr[2], fixef(Fm1)[2], fixef(Fm1)[3], fixef(Fm1)[4]), "\n")
    }
}

CalcModelsAge <- function(tis, dataDir, outDir) {
    # Loads the data and ensures data have the correct format
    # Args:
    #   tis: letter identifying the tissue to be run [F = fat, S = skin, L = LCLs,
B = blood]
    #   readsDir: directory for read counts outDir: directory for output
    # Returns:
    #   Variables ready to use for the RunModelsAge function

    reads <- read.table(file = paste(readsDir, "/EB_", tis,
"_normalize_exon_counts.txt",
        sep = ""), sep = "\t", header = T)
    covs <- read.table(file = paste(readsDir,"/covariates_", tis, ".txt",
        sep = ""), header = T, sep = "\t")

    family <- as.factor(as.matrix(covs["Family"]))
    zygosity <- as.factor(as.matrix(covs["Zygosity"]))
    insertSizeMode <- as.numeric(as.matrix(covs["INSERT_SIZE_MODE"]))
    gcMean <- as.numeric(as.matrix(covs["GC_mean"]))
    primerIndex <- as.factor(as.matrix(covs["PrimerIndex"]))
    date <- as.factor(as.matrix(covs["date"]))
    age <- as.numeric(as.matrix(covs["AGE"]))
    nIds <- dim(covs)[1] + 4
    #batch <- as.factor(as.matrix(covs['Set'])) # Use when running blood

    sink(file = paste(out_dir, "ExonsAgeAssociation_", tis, ".tab", sep = ""))
    RunModelsAge(reads, age, insertSizeMode, gcMean, primerIndex, date, zygosity,
family, nIds)
    sink()
}

# This runs the analysis by calling both functions
tis <- "F"
reads_dir <- ""
out_dir <- ""
CalcModelsAge(tis, dataDir, outDir)

# END 1.1)


# 1.2) Script to permute IDs keeping family structure.
# Date: 14/01/2013

Permutation <- function(n){
    # Get an index for each IDs, makes sure index are not repetead
    # Args:
    #   n: Number of individuals
    # Returns:
    #
   result = rep(0,n)
   used = rep(FALSE,n)
```

```
   for (i in 1:n){
     result[i] = which(!used)[as.integer(runif(1, 0, n-i+1) + 1)]
     used[result[i]] = TRUE
   }
   result
}

Get_permutation <- function(family, zygosity){
    # Permute the IDs of the individuals in groups. MZ twins are permuted only
with other
    # MZ twins, keeping the pair toguether. The same for DZ twins and unrelated
indviduals.
    # Unrelated individuals are those missing data for their sibling.
    # Args:
    #   family: index for family, as used in linear mixed models
    #   zygosity: index for zigosity, as used in linear mixed models
    # Returns:
    #   Individuals index permuted.

   perm = rep(0, length(family))

   # 0. determine who's who - unrelateds, mzs, dzs
   unrelateds = rep(FALSE, length(family))
   mzs = rep(FALSE, length(family))
   dzs = rep(FALSE, length(family))
   for (i in 1:length(family)){
     if (sum(family == family[i]) == 1){ unrelateds[i] = TRUE }
     else{
       if (as.character(zygosity[i]) == as.character(family[i])){ mzs[i] = TRUE }
# this is only for MZ; for DZ, zygosity = studyID
       else { dzs[i] = TRUE }
     }
   }

   # 1. permute unrelateds
   ur_I = Permutation(sum(unrelateds))
   perm[which(unrelateds)] = which(unrelateds)[ur_I]

   # 2. permute mz pairs
   mz_I = Permutation(sum(mzs)/2)
   families = unique(family[mzs])
   for (i in 1:(sum(mzs)/2)){ # for each pair
     for (j in 1:2){
       perm[which(family == families[i])[j]] = which(family == families[mz_I[i]])
[j]
     }
   }
   # 3. permute dzs like mzs
   dz_I = Permutation(sum(dzs)/2)
   families = unique(family[dzs])
   for (i in 1:(sum(dzs)/2)){ # for each pair
     for (j in 1:2){
       perm[which(family == families[i])[j]] = which(family == families[dz_I[i]])
[j]
     }
   }
   perm
}

# tis: letter identifying the tissue to be run [F = fat, S = skin, L = LCLs, B =
blood]
tis <- ""
covs = read.table(file = paste("covariates_", tis, ".txt", sep = ""), header = T,
sep = " ")
family <- as.factor(as.matrix(covs['Family']))
zygosity <- as.factor(as.matrix(covs['Zygosity']))
```

```
values_perm <- matrix(0,length(covs[,1]),150)
for(i in 1:100){ #This output 100 indexes permuted. Change the number if more are
need it
  values_perm[, i] <- as.vector(GetPermutation(family, zygosity))
}

# Export matrix with permuted index for use in further analysis
write.table(values_perm, file = paste("/", tis, "_permutedIDs.txt", sep = ""), sep
= "\t", quote = F, col.names = F, row.names = F)

## END 1.2)


# 1.3) Script to calculate age effect on gene expression with permuted IDs.

CalcModelsPerm <- function(tis, dataDir, outDir, permutation) {
    # Loads the data, permutations index and ensures data have the correct format
    # Args:
    #   tis: letter identifying the tissue to be run [F = fat, S = skin, L = LCLs,
B = blood]
    #   readsDir: directory for read counts
    #   outDir: directory for output
    #   permutation: index for the permutation (from 1 to 100)
    # Returns:
    #   Variables ready to use for the RunModelsAge function

    reads = read.table(file = paste(readsDir, "EB_", tis,
"_normalize_exon_counts.txt",
        sep = ""), sep = "\t", header = T)
    covs = read.table(file = paste(readsDir, "covariates_", tis, ".txt", sep =
""),
        header = T, sep = "\t")

    # A premutation file was previously generated (see 1.2). It has a column per
permutation,
    # with index from 1 to number of samples. Each column has a different, randomly
    # generated order for the samples index.
    perm = read.table(file = paste(readsDir, tis, "_permutedIDs.txt", sep = ""),
        sep = "\t", header = F)

    # From the permutation file, it selects the column corresponding to the
    # permutation index.
    Iperm <- as.numeric(perm[, permutation])

    family <- as.factor(as.matrix(covs["Family"]))
    zygosity <- as.factor(as.matrix(covs["Zygosity"]))
    insertSizeMode <- as.numeric(as.matrix(covs["INSERT_SIZE_MODE"]))
    gcMean <- as.numeric(as.matrix(covs["GC_mean"]))
    primerIndex <- as.factor(as.matrix(covs["PrimerIndex"]))
    date <- as.factor(as.matrix(covs["date"]))
    age <- as.numeric(as.matrix(covs["AGE"]))
    nIds <- dim(covs)[1] + 4
    #batch <- as.factor(as.matrix(covs['Set'])) # Use when running blood

    # This acutally output the results. It si faster than create a data.frame and
store it in memory
    sink(file = paste(out_dir, "Exons_Age_", tis, "_", permutation, ".tab", sep =
""))
    RunModelsPerm()
    sink()
}

RunModelsPerm <- function(reads, age, insertSizeMode, gcMean, primerIndex, date,
zygosity,
        family, nIds, Iperm) {
```

```
    # Computes the association between permuted expression and age using linear
mixed models
    # Args:
    #  I_perm: permuted index
    #  reads: data.frame with read counts per exon to be tested
    #  family: factor variable identifying families. This is coded with the family
ID (use same ID
    #    for sibllings)
    #  zygosity: factor variable identiying zigosity of individuals (MZ
    #    or DZ). Same number for MZ twins (family ID), different number for DZ
twins
    #  insertSizeMode: numeric variable describing insert size properties of the
    #    mRNAsequence
    #  gcMean: numeric variable describing mean values of GC content per
    #   sample
    #  primerIndex: factor variable describing primer index used for sequencing
    #  date: factor variable identifying samples sequenced the same date (a.k.a.
batch
    #    effect)
    #  age: numeric variable for the age of the individuals at the time of
    #   sample extraction
    #  n_ids: numeric variable with the number of individuals
    # Returns:
    #  A full text line with the fillowing columns: exonID, ChiSquare for
    # anova test, Pvalue for anova test, fix effect (beta) for age, fix effect
(beta)
    # for insertSizeMode, fix effect (beta) for gcMean.

    require(lme4)
    nReads <- dim(reads)[1]
    readsId <- NULL
    iReads <- NULL
    for (i_reads in 1:nReads) {
        readsId <- reads[iReads, c(1)]
        yIn = as.matrix(as.double(reads[iReads, 5:nIds][Iperm]))
        Fm1 = Fm2 = NULL

        Fm1 <- lmer(yIn ~ 1 + age + insertSizeMode + gcMean + (1 | primerIndex) +
            (1 | date) + (1 | zygosity) + (1 | family), REML = FALSE)
        Fm2 <- lmer(yIn ~ 1 + insertSizeMode + gcMean + (1 | primerIndex) + (1 |
            date) + (1 | zygosity) + (1 | family), REML = FALSE)

        cat(paste(as.character(reads[iReads, 1]), anova(Fm1, Fm2)$Chis[2], anova
(Fm1,
            Fm2)$Pr[2], fixef(Fm1)[2], fixef(Fm1)[3], fixef(Fm1)[4]), "\n")
    }
}

# This runs the analysis by calling both functions
tis <- "F"
reads_dir <- ""
out_dir <- paste("/", tis, "/", sep = "")

# The permutation index is taken from the job index. Submitting the script as an
# array allows to run the the same analysis as many times as permutations are
# requiered in semi-parallel jobs.
permutation = as.numeric(Sys.getenv("SGE_TASK_ID"))

CalcModelsPerm(tis, dataDir, outDir, permutation)

## END 1.3)


# 1.4) Script to calculate corrected p-value from permutations.

perm <- read.table("permuted.tab", sep = "\t", header = F) # File with the pvalues
```

```
from permuted data
tissue_summary <- read.table("association.results.tab", sep = "\t", header = F) #
File with nominal pvalues
# This tab delimited file has each gene ID in the first column, and the total
number of exons per gene in the second column
exons <- read.table(paste(tis, "_ExonsCount.txt", sep = ""), sep = "\t", header =
F)

permutation <- cbind(perm[, 1], perm[, 2:101])
# This object defines the groups used for the pvalues corrections
# Genes with similar number of exons within each range, are corrected toguether
groups <- cbind(c(seq(1, 10), seq(11, 20, 2), 21), c(seq(1, 10), seq(12, 20, 2),
    200))

all_pval <- NULL
pval_g <- NULL
genes_group <- NULL
perm_cluster <- NULL
fdr_group <- NULL
tissue_summary_group <- NULL
fdr_group <- NULL

# This loop runs for each one fo the 16 groups the correction of the pvalue based
on permuted pvalues.
for (i in 1:nrow(groups)) {
    genes_group <- exons[which(as.numeric(exons[, 2]) >= as.numeric(groups[i, 1])
&
        as.numeric(exons[, 2]) <= as.numeric(groups[i, 2])), 1]

    perm_cluster <- permutation[permutation[, 1] %in% genes_group, ]
    tissue_summary_group <- tissue_summary[tissue_summary[, 1] %in% genes_group, ]
    genes_group <- genes_group[genes_group %in% tissue_summary_group[, 1]]

# This loop runs each of the exons, correcting their pvalue
    fdr_group <- NULL
    for (j in 1:length(genes_group)) {
    # Here we count how many permuted pvalues are more significant that the
nominal pvalue.
    # The number divided by the total number of test performed, and divided by the
number of
    # permutaitons (perm = 100), provide the corrected pvalue
        fdr_group[j] <- (sum(perm_cluster[, 2:101] <= tissue_summary_group[j, 3])/
sum(tissue_summary_group[,
            3] <= tissue_summary_group[j, 3]))/100
    }
    # preparing output
    pval_g <- data.frame(genes_group, rep(i, length(fdr_group)), rep(length
(genes_group),
        length(fdr_group)), fdr_group)
    # Output: Exon ID, ID for the group, Num. of exon in group, corrected pvalue
    colnames(pval_g) <- c("ExonID", "Group", "ExonsInGroup", "CorrectedPvalue")
    all_pval <- rbind(all_pval, pval_g)
}

# Make sure file is order by exon ID.
all_pval <- all_pval[order(as.character(all_pval[, 1])), ]
# Add information from nominal pvalues
final_p_val <- cbind(tissue_summary, all_pval)
colnames(final_p_val) <- c("Exon", "ChiSq", "P_value", "Beta_Age",
"Beta_InsertSize",
    "Beta_GC", "ExonID", "Group", "ExonsInGroup", "FDR_correctedPvalue")
# Output matrix
write.table(final_p_val, "/home/anav/Eurobats/Results/
L_ExonsAgeingFDRcorrected.txt",
    sep = "\t", quote = F, row.names = F)
```

```
## END 1.4
# END 1


# 2) Scripts to calculate age effects on splicing of gene expression
# The same scripts is used for the expression association with age (analysis 1) is
used for the splicing analysis.
# The main difference is the phenotype used for splicing is the links (reads
spanning from one exon to another)
# identified by the software Altrans (Ongen & Dermitzakis, AJHG, 2015).
# END 2

# 3) Scripts to calculate age effect on variance of gene expression.
# 3.1) Scripts to calculate Residuals removing technical covariates and family
structure

# a)Square root of raw counts with a poisson distribution
tis <- "F"
reads = read.table(file = paste("EB_", tis, "_raw_counts.txt", sep = ""), sep =
"\t", header = T, row.names = NULL)
sqr.reads <- apply(reads[, 5:ncol(reads)], 2, sqrt) #square read counts to break
the mean-variance relationship
exons.names <- reads[, 1:4]
header = colnames(reads)
rm(tmp, reads)

# b) get residuals remove technical covariates and family structure + rank
normalize

Normal <- function(v){
    # Rank normalize the data
    # Args:
    #   v: vector with read counts per sample
    # Returns:
    #   vector with normaly distributed data
    w <- rep(0, length(v))
    ranking <- rank(v, ties.method="min")
    n <- length(unique(ranking))
    w[1:length(v)%in%ranking] <- qnorm(seq(from = 1 / (n + 1), to = n / (n + 1),
by = 1 / (n + 1)))
    return(w[ranking])
}

RunResiduals <- function(sqr.reads, age, insertSizeMode, gcMean, primerIndex,
date, zygosity,
        family, nIds){
  nReads <- dim(sqr.reads)[1]; readsId <- NULL ;
  require(lme4)
  iReads <- NULL
  for(iReads in 1:nReads){
    # Loading counts for one exon
    yIn = as.matrix(as.double(sqr.reads[iReads, 1:nIds]))
    Fm2 = NULL; y1 <- NULL
    # run linear mixed model with technical covariates and family relatiuonship
between samples
    Fm2 <- lmer(yIn ~ 1 + insertSizeMode + gcMean + (1 | primerIndex) + (1 | date)
+ (1 | zygosity) +
                (1 | family), REML = FALSE)
    sqr.reads.residuals <- residuals(Fm2) # Extract residuals
    y1 <- Normal(as.numeric(sqr.reads.residuals)) # Fix data distribution
    # Export data
    y2 <- c(as.character(exons.names[iReads, 1]), as.character(exons.names[iReads,
2]), as.character(exons.names[iReads, 3]),
                as.character(exons.names[iReads, 4]), round(y1, 10)) # Round
values to 10 decimals
```

```
    cat(paste(y2, sep = "\t"), '\n')
        }
}

# Here we read the covariates files and make sure the variables are the right data
type
covs = read.table(file = paste("covariates_",tis,".txt", sep = ""), header = T,
sep = "\t")

n_ids = dim(covs)[1]
family <- as.factor(as.matrix(covs["Family"]))
zygosity <- as.factor(as.matrix(covs["Zygosity"]))
insertSizeMode <- as.numeric(as.matrix(covs["INSERT_SIZE_MODE"]))
gcMean <- as.numeric(as.matrix(covs["GC_mean"]))
primerIndex <- as.factor(as.matrix(covs["PrimerIndex"]))
date <- as.factor(as.matrix(covs["date"]))
age <- as.numeric(as.matrix(covs["AGE"]))
nIds <- dim(covs)[1] + 4
#batch <- as.factor(as.matrix(covs['Set'])) # Use when running blood

sink(paste("EB_", tis, ".sqr.rank", sep = ""))
cat(paste(header), sep = "\t"), '\n')
RunResiduals(sqr.reads, age, insertSizeMode, gcMean, primerIndex, date, zygosity,
        family, nIds)
sink()

# END 3.1

# 3.2) Scripts to calculate age effect on variance of gene expression.

RunLoess <- function(){
    # Test for association between variance in gene expression and age
    # Args:
    #   residualS: data matrix with residuals after removing technical covariates
and
    #     individuals relationship using a linear mixed model
    #   age: age of the individuals
    # Returns:
    #   spearman correlation results for the association in text format
  nReads <- nrow(residualS)
  spear <- function(square.res,age) cor.test(square.res, age, exact = F, method =
"spearman")
  readsId <- NULL
  iIeads <- NULL
    for(iReads in 1:nReads) {
      yIn = as.matrix(as.double(residualS[iReads, 5:nCol]))
      #############################################
      square.res <- (loess(yIn ~ age)$residuals)^2
     ##############################################
        cat(paste(as.character(residualS[iReads, 1]), as.character(residualS
[iReads, 2]),
                as.character(residualS[iReads, 3]), as.character(residualS[iReads,
4]),
                spear(square.res, age)$p.value, spear(square.res, age)
$estimate) ,"\n")
        }
}

residualS = read.delim(file = paste("EB_", tis, ".sqr.rank", sep = ""), sep =
"\t", header = T)
covs = read.table(file = paste("covariates_", tis, "_freezev2.txt", sep = ""),
header = T, sep = "\t")

nCol = dim(covs)[1] + 4
age <- as.numeric(as.matrix(covs['AGE']))
```

```
sink(file = paste("Variance_", tis, "_Age.tab", sep = ""))
RunLoess(residualS, age, nCol)
sink()

# END 3.2

# 3.3) Scripts to permute IDs (no need to keep family structure, analysis on
# residuals)

# Since we are working with residuals, there is no need to maintain family
structure
# in the permutations.
tis = "F"
covs = read.table(file = paste("covariates_", tis, ".txt",sep = ""), header = T,
sep = "\t")
values_perm <- matrix(0, length(covs[, 1]), 100)

for(i in 1:100) {
  values_perm[, i] <- as.vector(sample(1:nrow(covs)))
}
write.table(values_perm, paste(tis, "_permutedIDs.txt", sep = "\t", quote = F,
col.names = F, row.names = F)


# 3.4) Script to run permutations

RunPermLoess <- function(Residuals, age, perm, n_col, n_perm){
    # Test for association between variance in gene expression and age
    #   using permuted values
    # Args:
    #   residualS: data matrix with residuals after removing technical covariates
and
    #     individuals relationship using a linear mixed model
    #   age: age of the individuals
    #   perm: matrix with permuted indexes for the samples
    # Returns:
    #   Pvalue for the spearman correlation results for the association in text
format
  spear <- function(square.res, age) cor.test(square.res, age, exact = F, method =
"spearman")
  readsId <- NULL
  i_reads <- NULL
    for(iReads in 1:nReads){
     yIn = as.matrix(as.double(residualS[iReads, 5:nCol]))
     #############################################
     square.res <- (loess(yIn ~ age)$residuals)^2
     ##############################################
     all.perm <- NULL
     for(iPerm in 1:nPerm){
      permAge <- as.numeric(perm[, iPerm])
      all.perm[iPerm] <- c(spear(square.res, permAge)$p.value)
      }
  cat(paste(c(as.character(residualS[iReads, 1]), all.perm)), "\n")
  }
}

# Load the data
nPerm = 100 # Choose number of permutaitons to run
tis <- "F"
data_dir = ""
out_dir = ""

residualS = read.delim(file = paste("EB_", tis, ".sqr.rank", sep = ""), sep =
"\t", header = T)
# Load covariates file
covs = read.table(file = paste("covariates_", tis, ".txt", sep = ""), header = T,
```

```
sep = "\t")
# Load permutaiton index and get age in the right numeric format
perm = read.table(file = paste(tis,"_permutedIDs.txt", sep = ""), sep = "\t",
header = F)
age <- as.numeric(as.matrix(covs['AGE']))

nReads <- nrow(residualS)
nCol = dim(covs)[1]+4

# Run the function and output the results as they are produced
sink(file = paste(out_dir, "Variance_", tis, "_AgePermuted.tab", sep = ""))
RunPermLoess(Residuals, age, perm, n_col, n_perm)
sink()

# END 3.4

# 3.5) Scripts to calculate FDR for variance

# It uses same script as 1.4

# END 3.5
# END 3

# 4) Scripts to calculate changes in discordance in gene expression with age.
# 4.1) Generate residuals removing technical covariates. Use only MZ twins with
siblings (full pairs)

Normal <- function(v){
    # Rank normalize the data
    # Args:
    #   v: vector with read counts per sample
    # Returns:
    #   vector with normaly distributed data
    w <- rep(0, length(v))
    ranking <- rank(v, ties.method="min")
    n <- length(unique(ranking))
    w[1:length(v)%in%ranking] <- qnorm(seq(from = 1 / (n + 1), to = n / (n + 1),
by = 1 / (n + 1)))
    return(w[ranking])
}

RunResiduals <- function(reads, age, insertSizeMode, gcMean, primerIndex, date,
zygosity,
        family, nIds){
  nReads <- dim(reads)[1]; readsId <- NULL ;
  require(lme4)
  iReads <- NULL
  for(iReads in 1:nReads){
    # Loading counts for one exon
    yIn = as.matrix(as.double(reads[iReads, 5:nIds]))
    Fm2 = NULL; y1 <- NULL
    # run linear mixed model with technical covariates
        Fm2 <- lmer(yIn ~ 1 + insertSizeMode + gcMean + (1 | primerIndex) + (1 |
date), REML = FALSE)
    reads.residuals <- residuals(Fm2) # Extract residuals
    y1 <- Normal(as.numeric(reads.residuals)) # Fix data distribution
    # Export data
    y2 <- c(as.character(exons.names[iReads, 1]), as.character(exons.names[iReads,
2]), as.character(exons.names[iReads, 3]),
                    as.character(exons.names[iReads, 4]), round(y1, 10)) # Round
values to 10 decimals
    cat(paste(y2, sep = "\t"), '\n')
        }
}

# Here we read the covariates files and make sure the variables are the right data
```

```
type
# This covariates file only have individuals with zygosity == MZ
# $ grep 'MZ' covariates_F.txt > covariates_F_MZ.txt
covs = read.table(file = paste("covariates_",tis,"_MZ.txt", sep = ""), header = T,
sep = "\t")

#To get only information from full pairs of MZ twins. Their familyIDs are the same
twin1 <- covs[duplicated(covs['Family']),1]
twin2 <- covs[!duplicated(covs['Family']),1]

nIds = dim(covs)[1]
insertSizeMode <- as.numeric(as.matrix(covs["INSERT_SIZE_MODE"]))
gcMean <- as.numeric(as.matrix(covs["GC_mean"]))
primerIndex <- as.factor(as.matrix(covs["PrimerIndex"]))
date <- as.factor(as.matrix(covs["date"]))
age <- as.numeric(as.matrix(covs["AGE"]))
nIds <- dim(covs)[1] + 4
#batch <- as.factor(as.matrix(covs['Set'])) # Use when running blood

# Get normally distributed residuals from the MZ pairs
sink(paste("EB_", tis, "_residualsMZ.rank", sep = ""))
cat(paste(header), sep = "\t"), '\n')
RunResiduals(reads, age, insertSizeMode, gcMean, primerIndex, date, nIds)
sink()

# END 4.1

# 4.2) Calculate discordance
# First we need to get per each twin pair the max and min expression values

tis = "S"

residualS = read.table(file = paste("EB_", tis, "_MZ.txt", sep = ""), sep = "\t",
header = T,
        stringsAsFactors = F)
covs = read.table(file = paste("covariates_", tis, "_MZ.txt", sep = ""), header =
T, sep = "\t")
nReads <- nrow(residualS)

#To get only information from full pairs of MZ twins. Their familyIDs are the same
twin1 <- covs[duplicated(covs['Family']), 1]
twin2 <- covs[!duplicated(covs['Family']), 1]

all.max <- NULL
all.min <- NULL
for(iReads in 1:nReads){
  MinTwinIds <- NULL
  MinTwin <- NULL
  MinTwinTmp <- NULL
  exonNames <- residualS[iReads, 1:4]
  exonValue <- cbind(as.numeric(residualS[iReads, colnames(residualS) %in%
twin1]),
        as.numeric(residualS[iReads, colnames(residualS) %in% twin2]))

  max.val <- cbind(exonNames, rbind(ifelse(exonValue[, 1] > exonValue[, 2],
exonValue[, 1], exonValue[, 2])))
  min.val <- cbind(exonNames, rbind(ifelse(exonValue[, 1] < exonValue[, 2],
exonValue[, 1], exonValue[, 2])))
  all.max <- rbind(all.max, max.val)
  all.min <- rbind(all.min, min.val)
}
write.table(all.max, paste("maxEB_", tis, "_MZ.txt", sep = ""), sep = "\t", quote
= F, row.names = F)
write.table(all.min, paste("minEB_", tis, "_MZ.txt", sep = ""), sep = "\t", quote
= F, row.names = F)
```

```
# Run association
all.max <- read.table(paste("maxEB_", tis, "_MZ.txt", sep = ""), sep = "\t", quote
= F, row.names = F)
all.min <- read.table(paste("minEB_", tis, "_MZ.txt", sep = ""), sep = "\t", quote
= F, row.names = F)

covs = read.table(file = paste("covariates_", tis, "_MZ.txt", sep = ""), header =
T, sep = "\t")

RunModelsDiscordant <- function(age, nCol, nReads, MaxTwinAll, MinTwinAll){
    # Run association between expression twin with maximun expression
    # and twin with minimum expresison depedent of age.
    # Args:
    #   age: numeric vector with age if the pair of twins
    #   nCol: number of columns
    #   nReads:  number of exons to run
    #   MaxTwinAll: normalized reads counts. The highest value per MZ pair
    #   MinTwinAll: normalized reads counts. The highest value per MZ pair
    # Returns:
    #   one line per exon with the following columns: Exon IDs, Gene ID,
    #   gene chromosome, gene TSS, beta value, SD, p-value
  iReads <- NULL
  for(iReads in 1:nrow(all.max)){
      MaxTwin1 <- as.numeric(as.matrix(all.max[iReads, 5:ncol(all.max)]))
    MinTwin1 <- as.numeric(as.matrix(all.min[iReads, 5:ncol(all.max)]))
      # This run  the actuall linear model
    Fm3 <- lm(MaxTwin1 ~ MinTwin1 + age)

      cat(paste(as.character(all.max[iReads, 1]), as.character(all.max[iReads,
2]), as.character(all.max[iReads, 3]),
                as.character(all.max[iReads, 4]), summary(Fm3)$coefficients[3, 1],
summary(Fm3)$coefficients[3, 2],
                summary(Fm3)$coefficients[3, 4]),"\n")
   }
}

sink(file=paste("/home/anav/Eurobats/Paper2/Discordance/
DiscondantTwins_Age_",tis,"_MZ.tab", sep=""))
RunModelsDiscordant(age, nCol, nReads, MaxTwinAll, MinTwinAll)
sink()

# END 4.2

# 4.3) Permutations and pvalues correction
#Use the same script as 1.4 and 1.5

# END 4.3
# END 4

# 5) Scripts to calculate genotype-by-age interactions effects on gene expression

# 5.1) Generate residuals removing thecnical covariates and family structure
# The residuals generation code is similar to the one described in 3, but using
# read counts.

Normal <- function(v){
    # Rank normalize the data
    # Args:
    #   v: vector with read counts per sample
    # Returns:
    #   vector with normaly distributed data
    w <- rep(0, length(v))
    ranking <- rank(v, ties.method="min")
    n <- length(unique(ranking))
    w[1:length(v)%in%ranking] <- qnorm(seq(from = 1 / (n + 1), to = n / (n + 1),
by = 1 / (n + 1)))
```

```
    return(w[ranking])
}

RunResiduals <- function(reads, age, insertSizeMode, gcMean, primerIndex, date, 
zygosity,
        family, nIds){
  nReads <- dim(reads)[1]; readsId <- NULL ;
  require(lme4)
  iReads <- NULL
  for(iReads in 1:nReads){
    # Loading counts for one exon
    yIn = as.matrix(as.double(sqr.reads[iReads, 1:nIds]))
    Fm2 = NULL; y1 <- NULL
    # run linear mixed model with technical covariates and family relatiuonship 
between samples
    Fm2 <- lmer(yIn ~ 1 + insertSizeMode + gcMean + (1 | primerIndex) + (1 | date) 
+ (1 | zygosity) +
                (1 | family), REML = FALSE)
    reads.residuals <- residuals(Fm2) # Extract residuals
    y1 <- Normal(as.numeric(reads.residuals)) # Fix data distribution
    # Export data
    y2 <- c(as.character(exons.names[iReads, 1]), as.character(exons.names[iReads, 
2]), as.character(exons.names[iReads, 3]),
                as.character(exons.names[iReads, 4]), round(y1, 10)) # Round 
values to 10 decimals
    cat(paste(y2, sep = "\t"), '\n')
        }
}

# Here we read the covariates files and make sure the variables are the right data 
type
covs = read.table(file = paste("covariates_",tis,".txt", sep = ""), header = T, 
sep = "\t")

n_ids = dim(covs)[1]
family <- as.factor(as.matrix(covs["Family"]))
zygosity <- as.factor(as.matrix(covs["Zygosity"]))
insertSizeMode <- as.numeric(as.matrix(covs["INSERT_SIZE_MODE"]))
gcMean <- as.numeric(as.matrix(covs["GC_mean"]))
primerIndex <- as.factor(as.matrix(covs["PrimerIndex"]))
date <- as.factor(as.matrix(covs["date"]))
age <- as.numeric(as.matrix(covs["AGE"]))
nIds <- dim(covs)[1] + 4
#batch <- as.factor(as.matrix(covs['Set'])) # Use when running blood

sink(paste("EB_", tis, ".residuals.rank", sep = ""))
cat(paste(header), sep = "\t"), '\n')
RunResiduals(reads, age, insertSizeMode, gcMean, primerIndex, date, zygosity,
        family, nIds)
sink()

# END 5.1

#5.2) Calculate associations of expression with an interaciton term of age and SNPs
# lm(x~age*SNP)


getResults <- function(reads, age, nIds, gene.pos, dosage.chr, snp.pos){
    # Runs a linear regressions including an interaction between genotype (SNP)
    # and age.
    # Args:
    #   reads: residuals for gene expression
    #   age: vector with the age of the individuals
    #   nIDs: number of individuals
    #   gene.pos: TSS of the gene
    #   dosage.chr: dosage values for the chromosome of the gene
```

```
    #   snp.pos: position of the SNPs in the chomosome
    # Returns:
    #   One line per exon~SNP assocation with Exon ID, SNP ID, Pvalue, beta, SD

  nReads <- dim(reads)[1]
  iReads <- NULL; gene.TSS <- NULL; cis.snp <- NULL
  cis.dosage <- NULL;exon.name <- NULL; iSnp <- NULL

    for(iReads in 1:nReads){
      exon.name <- as.character(reads[iReads, 1])
      yIn = as.matrix(as.double(reads[iReads, 2:nIds]))

      # From the gene TSS, get all SNPs around for cis analysis
      gene.TSS <- gene.pos[gene.pos[, 1] %in% reads[iReads, 1], 3]
      cis.snp <- snp.pos[which(snp.pos[, 3] > gene.TSS - (1e+6) & snp.pos[, 3] <
gene.TSS
                + (1e+6)), 1]
      cis.dosage <- dosage.chr[dosage.chr[, 1] %in% cis.snp, ]

      for(iSnp in 1:nrow(cis.dosage)){
                if(length(cis.snp) == 0) { next # In case an exon doesn't have
SNPs in cis window
                        } else {

       Fm1 <- NULL; SNP <- NULL; snp.name <- NULL
       snp.name <- as.character(cis.dosage[iSnp, 1])
       SNP = as.matrix(as.double(cis.dosage[iSnp, 2:nIds]))
           # This runs the association
       p <- summary(lm(yIn ~ age*SNP))
       # Output is Exon ID, SNP ID, Pvalue, beta, SD
       p2 <- c(exon.name, snp.name, p$coeff[4, 4], p$coeff[4, 1], p$coeff[4, 2])
       cat(paste(p2), "\n")
      }
    }
  }
}

# Load files
tis <- ""  # Tissue
residualsDir <- paste("/") #Directory with the residuals and genotypes
readsDir <-  paste("/") #Directory with the residuals and genotypes
outDir <- "/" # Output directory

#Job array ID: 50 genes per array job, with all their exons
index = as.numeric(Sys.getenv("SGE_TASK_ID"))

# It need a file with a list of exons, the TSS for the gene and a run index.
# The run index is the same for groups of 50 genes with all their exons.

gene.list = read.table(file = paste(residualsDir, "/", tis, "_exon_list.txt", sep
= ""), sep="\t", header=F)
l <- sapply(1:nrow(gene.list), function(x) paste(gene.list[x, 1], gene.list[x, 2],
sep = ','))
gene.info <- unique(gene.list[l == unique(l)[index], ])# Genes and TSS for the 50
genes to run

# File with the residuals were also split in groups of 50 genes as indicated in
the gene.list object.
reads = read.table(file = paste(residuals_dir, "/", "Residuals_", tis, "_chr",
gene.info[1, 1], ".matrix",
        sep = ""), sep = "\t", header = F)
reads = reads[substr(reads[, 1], 1, 15) %in% gene.info[, 3], ] #select residuals
from 50 genes to run

# covariates file
covs = read.table(file = paste(readsDir, "/", "covariates_", tis, "_freezev1.txt",
```

```
sep = ""), header = T, sep = "\t")
gene.pos = read.table(file=paste(residualsDir,"/","chr",gene.info
[1,1],"_",tis,"_genes.locations.matrix",
        sep = ""), sep = "\t", header = F) # get gene locations
dosage.chr = read.table(file = paste(residualsDir, "/", tis, "_chr", gene.info[1,
1], "_", gene.info[1, 2],
        ".dosage.maf5", sep = ""), sep = "\t", header = F, skip = 1)# dosage files
from genotypes
snp.pos = read.table(file = paste(residualsDir, "chr", gene.info[1, 1],
"_snppos.matrix.maf5", sep = ""),
        sep = "\t",     header = T)# SNPs positions

age <- as.numeric(as.matrix(covs['AGE'])) # for Fat, Skin and LCLs
nIds = dim(covs)[1]+1

# This one runs the analysis
sink(file=paste(out_dir,"chr",gene.info[1,1],"_",gene.info
[1,2],"_",tis,"_GxA_LM.tab", sep=""))
GetResults(reads, age, nIds, gene.pos, dosage.chr, snp.pos)
sink()

# END 5.2

#5.3) Permutations
# Permutations are based in Gerrits et al Plos Genetics, 2009.

Regress <- function(num.perm, y_in, SNP, age, perm){
    # This is a modified version of lm() for linear regressions. In order to speed
    # up the analysis we only invert the matrix once.
    # WARNING! this may fails if the linear model is unstable.
    # Args:
    #   SNP: vector with dosages values for the genotype to test
    #   age: vector with the age of the individuals
    #   yIn: expression values per exon (residuals)
    #   num.perm: index for the permutaiton run
    #   perm: matrix with permuted indexes
    # Returns:
    #   vector with pvalues, the first column is the nominal pvalue.

  model <- cbind(rep(1, length(SNP)), SNP, age) # Get variable for the simple model
  # Get residuals for a model without interaction term
  yInRes <- yIn - model%*% solve(crossprod(model), crossprod(model, yIn))

  model <- cbind(model, SNP*age) # Get variable for a full model with interactions
  invert <- solve(crossprod(model)) # inver the matrix
  # Get residuals from simple model and permuted them according to permuted indexes
  y.in.perm <- matrix(yInRes[unlist(perm[, 1:num.perm])], nrow(perm), num.perm)
  # Inver matrix
  beta <- invert%*%crossprod(model, y.in.perm)
  # Calculate manually pvalues from the linear regression.
  p.values <- signif(2*pt(-abs(beta[4, ]/sqrt(colSums((y.in.perm - model%*%beta) ^
2) /
        (nrow(model) - 4) * invert[4, 4])), nrow(model) - 4), digits = 10)
}

RegressFailed <- function(num.perm, y_in, SNP, age, perm){
    # Runs the linear association using the function lm() in R.
    # Use it if the Regress() function fails
    # Args:
    #   SNP: vector with dosages values for the genotype to test
    #   age: vector with the age of the individuals
    #   yIn: expression values per exon (residuals)
    #   num.perm: index for the permutaiton run
    #   perm: matrix with permuted indexes
    # Returns:
    #   vector with pvalues, the first one is the nominal pvalue.
```

```
  yInRes <- residuals(lm(yIn ~ age + SNP))
  y.in.perm <- matrix(yInRes[unlist(perm[, 1:num.perm])], nrow(perm[, 1:num.perm]),
ncol(perm[, 1:num.perm]))
  p.values <- signif(apply(y.in.perm, 2, function(x)summary(lm(x ~ age * SNP))
$coeff[4, 4]), digits = 10)
}

GetInteraction <- function(reads, age, nIds, gene.pos, dosage.chr, snp.pos,
runPerm, num.perm){
  nReads <- dim(reads)[1]
  iReads <- NULL
  gene.TSS <- NULL
  cis.snp <- NULL
  cis.dosage <- NULL
  exon.name <- NULL
  iSnp <- NULL
  for(iReads in 1:nReads){
    exon.name <- as.character(reads[iReads, 1])
    yIn = as.matrix(as.double(reads[iReads, 5:ncol(reads)]))

    gene.TSS <- gene.pos[gene.pos[, 1] %in% reads[iReads, 1], 3]
    cis.snp <- snp.pos[which(snp.pos[, 3] > gene.TSS - (1e+6) & snp.pos[, 3] <
gene.TSS+(1e+6)), 1]
    cis.dosage <- dosage.chr[dosage.chr[, 1] %in% cis.snp, ]

    for(i_snp in 1:nrow(cis.dosage)) {
            if(length(cis.snp) == 0){next # In case an exon doesn't have SNPs
in cis window
        } else {
            Fm1 <- NULL; SNP <- NULL; snp.name <- NULL
        snp.name <- as.character(cis.dosage[iSnp, 1])

        SNP = as.matrix(as.double(cis.dosage[i_snp, 2:nIds]))
        # Get residuals, permute them and run permutation
        # This calls a function which returns num.perm permuted p values.
        # This function can fail if things are co-linear, in which case the
tryCatch will do it with lm()
        perm.p <- tryCatch(Regress(num.perm, yIn, SNP, age, perm), error = function
(x) RegressFailed(num.perm, yIn, SNP, age, perm))
        perm.p2 <- c(exon.name, snp.name, perm.p)
        cat(paste(perm.p2), "\n")
        }
    }
  }
}

# Load files
tis <- ""  # Tissue
residualsDir <- "/" #Directory with the residuals and genotypes
readsDir <- "/" #Directory with the residuals and genotypes
# Number of permutations to run. The final oputput will have one column for exon,
one for SNP and
#       as many colum ns as permuted p-values asked here.
num.perm <- 11
outDir <- "/" # Output directory

index = as.numeric(Sys.getenv("SGE_TASK_ID"))

gene.list = read.table(file=paste(residuals_dir,tis,"_exon_list.txt", sep=""),
sep="\t", header=F)
l <- sapply(1:nrow(gene.list),function(x)paste(gene.list[x,1],gene.list
[x,2],sep=','))
gene.info <- unique(gene.list[l==unique(l)[index],])

#  reads = read.table(file=paste(residuals_dir,"Residuals_",tis,"_chr",gene.info
```

```
[1,1],"_",gene.info[1,2],sep=""), sep="\t", header=F)
reads = read.table(file=paste(residuals_dir,"/chr",gene.info
[1,1],"_",tis,"_TechCovFam_2014_freezev1.rank",sep=""), sep="\t", header=F)
reads = reads[substr(reads[,1],1,15)%in% gene.info[,3],]

covs = read.table(file = paste(readsDir, "/", "covariates_", tis, "_freezev1.txt",
sep = ""), header = T, sep = "\t")
gene.pos = read.table(file=paste(residualsDir,"/","chr",gene.info
[1,1],"_",tis,"_genes.locations.matrix",
        sep = ""), sep = "\t", header = F) # get gene locations
dosage.chr = read.table(file = paste(residualsDir, "/", tis, "_chr", gene.info[1,
1], "_", gene.info[1, 2],
        ".dosage.maf5", sep = ""), sep = "\t", header = F, skip = 1)# dosage files
from genotypes
snp.pos = read.table(file = paste(residualsDir, "chr", gene.info[1, 1],
"_snppos.matrix.maf5", sep = ""),
        sep = "\t",     header = T)# SNPs positions
perm = read.table(file=paste(residuals_dir,tis,"_permutedIDs.txt", sep=""),
sep="\t", header=F)

age <- as.numeric(as.matrix(covs['AGE']))
nIds = dim(covs)[1]+1

# This one runs the analysis + permutations
sink(file=paste(out_dir,"Perm_chr",gene.info[1,1],"_",gene.info
[1,2],"_",tis,"_Interactions_exons.tab", sep=""))
GetInteraction(reads, age, n_ids, gene.pos,dosage.chr,snp.pos,run_perm, num.perm)
sink()

# END 5.3

#5.4) Corrected Pvalues
# It uses the same script as in 1.4 and 1.5

# END 5.4
## END 5

# 6) Scripts to perform methylation associations analysis.

# 6.1) Methylation associations with age a) Association b) Permutation c) Pvalue
# correction

# Methylation ~ age
runModelsAge <- function(family, zygosity, bscConc, bscEfficiency, chip, age,
        nIdsMethy, methy) {
    # Computes the association between methylation and age using linear mixed
models
    # Args:
    #  methy: data.frame with methylation levels be tested
    #  family: factor variable identifying families. This is coded with the family
ID (use same ID
    #    for sibllings)
    #  zygosity: factor variable identiying zigosity of individuals (MZ
    #    or DZ). Same number for MZ twins (family ID), different number for DZ
twins
    #  bscConc: BS-treated DNA input
    #  bscEfficiency: BS conversion efficiency
    #  chip: beadchip type
    #  age: numeric variable for the age of the individuals at the time of
    #   sample extraction
    # Returns:
    #  A full text line with the fillowing columns: methylation marker ID,
ChiSquare for
    # anova test, Pvalue for anova test, fix effect (beta) for age, fix effect
(beta)
    # for bscConc, fix effect (beta) for bscEfficiency and  fix effect (beta) for
```

```
chip.
   require(lme4)
   require(Matrix)
   iMethy <- NULL
   for(iMethy in 1:nrow(methy)) {

     Fm1 <- NULL; Fm2 <- NULL;
     site.methy <- as.matrix(as.double(methy[iMethy, 2:nIdsMethy]))

     Fm1 <- lmer(site.methy ~ 1 + age + bscConc + bscEfficiency + chip + (1 |
zygosity) + (1 | family),
                REML = FALSE)
     Fm2 <- lmer(site.methy ~ 1 + bscConc +  bscEfficiency + chip + (1 | zygosity)
+ (1 | family),
                REML = FALSE)

        cat(paste(as.character(methy[i_methy, 1]), anova(Fm1, Fm2)$Chis[2], anova
(Fm1, Fm2)$Pr[2],
                fixef(Fm1)[2], fixef(Fm1)[3], fixef(Fm1)[4], fixef(Fm1)[5]), "\n")
   }
}


outDir <- "/"
outDir <- "/"

# The analysis runs chromosome by chromosome for speed
index = as.numeric(Sys.getenv("SGE_TASK_ID")) #23

methy <- read.table(paste("EB_F_chr", index, ".txt", sep = ""), sep = "\t", header
= F,
        colClasses = c(rep("character", 1), rep("numeric", 516)))

# Individuals information (family and zygosity)
covs = read.table(file = paste("Covariates_F_methylation.txt", sep = ""), header =
T, sep = "\t")
# Techncial covariates for methylaiton array
covs.methy = read.table(file = paste("info_450kfat_EB_menarche.txt", sep = ""),
header = T, sep = "\t")
# Information about location of CpGs reported in the methylation array
info.methy = read.table(file = paste("A-MEXP-2255.info.menarche.txt", sep = ""),
header = T, sep = "\t")

family <- as.factor(as.matrix(covs['Family']))
zygosity <- as.factor(as.matrix(covs['Zygosity']))
bsc_efficiency <- as.numeric(as.matrix(covs.methy['bsc_efficiency_22795447']))
bsc_conc <- as.numeric(as.matrix(covs.methy['bsc_conc']))
chip <- as.factor(as.matrix(covs.methy['Chip']))
age <- as.numeric(as.matrix(covs.methy['age']))

nIds_reads = dim(covs)[1]+4
nIds_methy = dim(covs)[1]+1

sink(file = paste(out_dir, "F_MethylationAge_chr", index, ".output", sep = ""))
RunModelsAge(family, zygosity, bscConc, bscEfficiency, chip, age, nIdsMethy, methy)
sink()

# END 6.1

# 6.2) Calculate permuted pvalue for metahylation ~ age association.

RunModelsAgePerm <- function(family, zygosity, bscEfficiency, bscConc, chip, age,
        nIdsMethy, methy, info.methy, perm, gene.info){
    # Computes the association between permuted methylation and age using linear
mixed models
    # Args:
```

```
    #  methy: data.frame with methylation levels be tested
    #  family: factor variable identifying families. This is coded with the family
ID (use same ID
    #    for sibllings)
    #  zygosity: factor variable identiying zigosity of individuals (MZ
    #    or DZ). Same number for MZ twins (family ID), different number for DZ
twins
    #  bscConc: BS-treated DNA input
    #  bscEfficiency: BS conversion efficiency
    #  chip: beadchip type
    #  age: numeric variable for the age of the individuals at the time of
    #   sample extraction
    #   perm: table with the indexes permuted
    # Returns:
    #  A full text line with the methylation region ID and 10 pvalues prduced from
        #  permuted pvalues.

 require(lme4)
 require(Matrix)
 permutations = 10

 if(index == 23){ # Chromsome 23 is chromosome X in the information file.
   tmp <- info.methy[which(info.methy[,7] == "chr23"),]
   }else{tmp <- info.methy[info.methy[, 7] %in% paste("chr", gene.info[1, 1], sep
= ""), ]
 }

 iMethy <- NULL
 for(iMethy in 1:nrow(methy)) {

   pval <- rep(NA, permutations)
   for(p in 1:permutations) {
    Fm1 <- NULL; Fm2 <- NULL;
        Iperm <- as.numeric(perm[, p])
        # Permute methylation level based on permuted indexes
    site.methy <- as.matrix(as.double(methy[iMethy, 2:nIdsMethy][I_perm]))

    Fm1 <- lmer(site.methy ~ 1 + age + bscConc +  bscEfficiency + chip + (1 |
zygosity) +
                (1 | family), REML = FALSE)
    Fm2 <- lmer(site.methy ~ 1 + bscConc +  bscEfficiency + chip + (1 | zygosity)
+
                (1 | family), REML = FALSE)
        pval[p] <- c(as.numeric(anova(Fm1, Fm2)$Pr[2]))
    }
  # Run each of the 10 permutatins in each run.
  cat(paste(as.character(methy[iMethy, 1]), pval[1], pval[2], pval[3], pval[4],
pval[5],
        pval[6], pval[7], pval[8], pval[9], pval[10]), "\n")
  }
}

outDir <- "/"

permutations = 10  # Number of permutations to perform
perm = read.table(file = "Covariates_F_permuted_methylation.txt", sep = "\t",
header = F)

# The analysis runs once chromosme per permutation
# Files can be splited more if too splow.
index = as.numeric(Sys.getenv("SGE_TASK_ID")) #10

methy <- read.table(paste("EB_F_chr", index, ".txt", sep = ""), sep = "\t", header
= F,
        colClasses = c(rep("character", 1), rep("numeric", 516)))
```

```
# Individuals information (family and zygosity)
covs = read.table(file = paste("Covariates_F_methylation.txt", sep = ""), header =
T, sep = "\t")
# Techncial covariates for methylaiton array
covs.methy = read.table(file = paste("info_450kfat_EB_menarche.txt", sep = ""),
header = T, sep = "\t")
# Information about location of CpGs reported in the methylation array
info.methy = read.table(file = paste("A-MEXP-2255.info.menarche.txt", sep = ""),
header = T, sep = "\t")

family <- as.factor(as.matrix(covs['Family']))
zygosity <- as.factor(as.matrix(covs['Zygosity']))
bscEfficiency <- as.numeric(as.matrix(covs.methy['bsc_efficiency_22795447']))
bscConc <- as.numeric(as.matrix(covs.methy['bsc_conc']))
chip <- as.factor(as.matrix(covs.methy['Chip']))
age <- as.numeric(as.matrix(covs.methy['age']))

nIds_reads = dim(covs)[1]+4
nIds_methy = dim(covs)[1]+1

sink(file = paste(out_dir, "F_MethylationAge_chr", index, ".output", sep = ""))
RunModelsAgePerm(family, zygosity, bscConc, bscEfficiency, chip, age, nIdsMethy,
methy)
sink()

# END 6.2

# 6.3 Pvalues correction.
# Use same analysis as 1.4 and 1.5 to correct pvalues using pemruted pvalues

# END 6.3


# 6.4) Calculate expression-methylation association
# Expresison values need to be corrected for technical covariates and family
structure
# The analysis is run useing residuals generated as decribed in analysis 3.1.

runMethyExp <- function(reads, family, zygosity, bscConc, bscEfficiency, chip,
nIdsReads,
        nIdsMethy, methy, covs.methy, info.methy, gene.info) {
    # Computes the association between methylation and expression using linear
mixed models
    # Args:
    #  reads: data.frame with residuals from expression
    #  methy: data.frame with methylation levels be tested
    #  family: factor variable identifying families. This is coded with the family
ID (use same ID
    #    for sibllings)
    #  zygosity: factor variable identiying zigosity of individuals (MZ
    #    or DZ). Same number for MZ twins (family ID), different number for DZ
twins
    #  bscConc: BS-treated DNA input
    #  bscEfficiency: BS conversion efficiency
    #  chip: beadchip type
    # Returns:
    #  A full text line with the following columns: methylation marker ID,
ChiSquare for
    # anova test, Pvalue for anova test, fix effect (beta) for age, fix effect
(beta)
    # for bscConc, fix effect (beta) for bscEfficiency and  fix effect (beta) for
chip.

  nReads <- dim(reads)[1]
  readsId <- NULL
  require(lme4)
```

```
  require(Matrix)
  iReads <- NULL
  for(iReads in 1:nrow(reads)) {
    expr = as.matrix(as.double(reads[iReads, 2:nIdsReads]))
    Fm1 = Fm2 = NULL

    info <- gene.info[gene.info[, 3] %in% substr(reads[iReads, 1], 1, 15), ]
    if(info[, 1] == 23) {
          tmp <- info.methy[which(info.methy[, 7] == "chr23"), ]
      }else{tmp <- info.methy[info.methy[, 7] %in% paste("chr", info[, 1], sep =
""), ]
    }

    sites <- tmp[which(tmp[, 8] < (info[, 4] + (50000)) & tmp[, 8] > (info[, 4] -
(50000))), ]
    values.methy <- methy[methy[, 1] %in% sites[, 5], ]

    if(nrow(values.methy) != 0){ #make sure there is CpGs in the cis window.
      iMethy <- NULL
      for(iMethy in 1:nrow(values.methy)) {
        Fm1 <- NULL; Fm2 <- NULL;
        site.methy <- as.matrix(as.double(values.methy[iMethy, 2:nIdsMethy]))
        Fm1 <- lmer(site.methy ~ 1 + expr + age + bscConc +  bscEfficiency + chip
+  (1 | zygosity)
                        + (1 | family), REML = FALSE)
        Fm2 <- lmer(site.methy ~ 1 + age + bscConc +  bscEfficiency + chip  + (1 |
zygosity) +
                        (1 | family), REML = FALSE)
                cat(paste(as.character(reads[iReads, 1]), as.character(values.methy
[iMethy, 1]), anova(Fm1, Fm2)$Chis[2],
                        anova(Fm1, Fm2)$Pr[2], fixef(Fm1)[2], fixef(Fm1)[3], fixef
(Fm1)[4], fixef(Fm1)[5]), "\n")
        }
    }
  }
}

outDir <- "/"
# Files are splited in 200 CpGs per file.
index = as.numeric(Sys.getenv("SGE_TASK_ID"))#394

# File with indexes to run groups of genes
gene.list = read.table(file="exon_list.txt", sep="\t", header=F)
l <- sapply(1:nrow(gene.list),function(x)paste(gene.list[x,1],gene.list
[x,2],sep=','))
gene.info <- unique(gene.list[l==unique(l)[index],])
chr <- unique(gene.info[,1])

methy <- read.table(paste("/home/avinuela/nqs/Methylation/chr",gene.info
[1,1],"_",gene.info[1,2],".info", sep=""), sep="\t", header=F,  colClasses =c(rep
("character",1),rep("numeric",516)))
covs.methy = read.table(file=paste("/home/avinuela/nqs/Methylation/
info_450kfat_EB_menarche.txt",sep=""),header=T,sep="\t")
info.methy = read.table(file=paste("/home/avinuela/nqs/Methylation/A-
MEXP-2255.info.menarche.txt",sep=""),header=F,sep="\t")

reads = read.table(file = paste("Residuals_Expression.tab", sep = ""), sep = "\t",
header = T)
# Select the exons from genes included in the region
reads <- reads[substr(reads[, 1], 1, 15) %in% gene.info[, 3], ]

# Individuals information (family and zygosity)
covs = read.table(file = paste("Covariates_F_methylation.txt", sep = ""), header =
T, sep = "\t")
# Techncial covariates for methylaiton array
covs.methy = read.table(file = paste("info_450kfat_EB_menarche.txt", sep = ""),
```

```
header = T, sep = "\t")
# Information about location of CpGs reported in the methylation array
info.methy = read.table(file = paste("A-MEXP-2255.info.menarche.txt", sep = ""),
header = T, sep = "\t")

family <- as.factor(as.matrix(covs['Family']))
zygosity <- as.factor(as.matrix(covs['Zygosity']))
bscEfficiency <- as.numeric(as.matrix(covs.methy['bsc_efficiency_22795447']))
bscConc <- as.numeric(as.matrix(covs.methy['bsc_conc']))
chip <- as.factor(as.matrix(covs.methy['Chip']))

nIds_reads = dim(covs)[1]+4
nIds_methy = dim(covs)[1]+1

sink(file=paste(outDir,"F_MethylationExpression_chr",gene.info[1,1],"_",gene.info
[1,2],".output",sep=""))
runMethyExp(reads, family, zygosity, bscConc, bscEfficiency, chip, nIdsReads,
nIdsMethy,
        methy, covs.methy, info.methy, gene.info)
sink()

# END 6.4

# 6.5) Permutation


runMethyExpPerm <- function(reads, family, zygosity, bsc_conc, bsc_efficiency,
chip, n_ids_reads, n_ids_methy , methy, covs.methy, info.methy, gene.info,
perm,indexPerm){
    # Computes the association between methylation and expression using linear
mixed models
    # Args:
    #  reads: data.frame with residuals from expression
    #  methy: data.frame with methylation levels be tested
    #  family: factor variable identifying families. This is coded with the family
ID (use same ID
    #    for sibllings)
    #  zygosity: factor variable identiying zigosity of individuals (MZ
    #    or DZ). Same number for MZ twins (family ID), different number for DZ
twins
    #  bscConc: BS-treated DNA input
    #  bscEfficiency: BS conversion efficiency
    #  chip: beadchip type
    # Returns:
    #  A full text line with the following columns: methylation marker ID,
ChiSquare for
    # anova test, Pvalue for anova test, fix effect (beta) for age, fix effect
(beta)
    # for bscConc, fix effect (beta) for bscEfficiency and  fix effect (beta) for
chip.
 nReads <- dim(reads)[1]
 readsId <- NULL
 require(lme4)
 require(Matrix)
 iReads <- NULL
 for(iReads in 1:nrow(reads)) {
  expr = as.matrix(as.double(reads[iReads, 5:nIdsReads]))
  Fm1 = Fm2 = NULL

  info <- gene.info[gene.info[, 3] %in% substr(reads[iReads, 1], 1, 15), ]
  if(info[, 1] == 23) {
    tmp <- info.methy[which(info.methy[, 7] == "chr23"), ]
    }else{tmp <- info.methy[info.methy[, 7] %in% paste("chr", info[, 1], sep =
""), ]
  }
```

```
  sites <- tmp[which(tmp[, 8] < (info[, 4] + (50000)) & tmp[, 8] > (info[, 4] -
(50000))), ]
  values.methy <- methy[methy[, 1] %in% sites[, 5], ]

  if(nrow(values.methy) != 0) {
  iMethy <- NULL
   for(iMethy in 1:nrow(values.methy)) {
     pval <- rep(NA, permutations)
     Fm1 <- NULL; Fm2 <- NULL;
     iPerm <- as.numeric(perm[, indexPerm])
     site.methy <- as.matrix(as.double(methy[iMethy, 2:n_ids_methy][iPerm]))

     Fm1 <- lmer(site.methy ~ 1 + expr + age + bscConc +  bscEfficiency + chip +
(1 | zygosity)
                + (1 | family), REML = FALSE)
     Fm2 <- lmer(site.methy ~ 1 + age + bscConc +  bscEfficiency + chip  + (1 |
zygosity) +
                (1 | family), REML = FALSE)
     pval <- c(as.numeric(anova(Fm1,Fm2)$Pr[2]))
         cat(paste(as.character(reads[iReads,1]), as.character(methy[iMethy, 1]),
pval[1], "\n"))
    }
   }
  }
}

outDir <- "/"
# Files are splited in 200 CpGs per file.
index = as.numeric(Sys.getenv("SGE_TASK_ID"))#394

indexPerm = 1
permutations = 1
perm = read.table(file = "Covariates_F_permuted_methylation", sep = "\t", header =
F)

# File with indexes to run groups of genes
gene.list = read.table(file="exon_list.txt", sep="\t", header=F)
l <- sapply(1:nrow(gene.list),function(x)paste(gene.list[x,1],gene.list
[x,2],sep=','))
gene.info <- unique(gene.list[l==unique(l)[index],])
chr <- unique(gene.info[,1])

methy <- read.table(paste("chr", gene.info[1, 1], "_", gene.info[1, 2], ".info",
sep = ""), sep = "\t",
      header = F,  colClasses = c(rep("character", 1), rep("numeric", 516)))
covs.methy = read.table(file = paste("info_450kfat_EB.txt", sep = ""), header = T,
sep = "\t")
info.methy = read.table(file = paste("A-MEXP-2255.info.txt", sep = ""), header =
F, sep = "\t")

reads = read.table(file = paste("Residuals_Expression.tab", sep = ""), sep = "\t",
header = T)
# Select the exons from genes included in the region
reads <- reads[substr(reads[, 1], 1, 15) %in% gene.info[, 3], ]

# Individuals information (family and zygosity)
covs = read.table(file = paste("Covariates_F_methylation.txt", sep = ""), header =
T, sep = "\t")
# Techncial covariates for methylaiton array
covs.methy = read.table(file = paste("info_450kfat_EB_menarche.txt", sep = ""),
header = T, sep = "\t")
# Information about location of CpGs reported in the methylation array
info.methy = read.table(file = paste("A-MEXP-2255.info.menarche.txt", sep = ""),
header = T, sep = "\t")

family <- as.factor(as.matrix(covs['Family']))
```

```
zygosity <- as.factor(as.matrix(covs['Zygosity']))
bscEfficiency <- as.numeric(as.matrix(covs.methy['bsc_efficiency_22795447']))
bscConc <- as.numeric(as.matrix(covs.methy['bsc_conc']))
chip <- as.factor(as.matrix(covs.methy['Chip']))

nIds_reads = dim(covs)[1]+4
nIds_methy = dim(covs)[1]+1


sink(file = paste(outDir, "F_MethylationExpression_chr", gene.info[1, 1], "_",
gene.info[1, 2], ".perm_", indexPerm, sep = ""))
runMethyExpPerm(reads, family, zygosity, bscConc, bscEfficiency, chip, nIdsReads,
nIdsMethy,
        methy, covs.methy, info.methy, gene.info, perm, indexPerm)
sink()

# END 6.5

# 6.6) Pvalue correction
# Run similar analysis as 1.4 and 1.5

# END 6.6
# END 6
```
